# Knowledge-seeking and knowledge sharing of health services across social networks and communities: a scoping review

**DOI:** 10.1186/s12913-025-12525-y

**Published:** 2025-03-27

**Authors:** Anamitra Bhowmick, Marieke M. van der Zande, Rebecca Harris

**Affiliations:** Department of Public Health, Policy and Systems, Institute of Population Health, University of Liverpool, Whelan Building, Liverpool, L69 3GL UK

**Keywords:** Healthcare knowledge, Social network, Health service utilisation, Health literacy

## Abstract

**Introduction:**

Lay people’s knowledge influences healthcare service utilisation, but the literature on people’s knowledge-seeking and sharing about different healthcare services across social networks is patchy and not well integrated. This scoping review was undertaken to map how different studies report healthcare service -related (healthcare) knowledge-seeking or sharing in social circles and to identify evidence gaps for further research.

**Method:**

Levac’s enhanced scoping review framework was adapted to develop a comprehensive electronic search strategy. Four electronic databases-Medline, Web of Science, PsychINFO, and CINAHL were searched as well as Grey literature. Five per cent of all titles and abstracts screened were screened by a blinded second reviewer. After full-text screening, data were extracted and summarised.

**Results:**

The review included 14 quantitative, 23 qualitative, 2 mixed-method studies, one literature review and one report [*N* = 41]. Theories included within studies ranged from the socio-ecological model to bricolage. The concept of healthcare-related knowledge was generally ill defined and usually positioned within the concept of health literacy more generally. Lay people’s healthcare knowledge was not generally considered as a distinct entity in a holistic sense, with only two studies identified which investigated healthcare knowledge exclusively at inter-personal (meso) levels. However, included studies showed that people’s healthcare knowledge in everyday life is co-constructed when they engage in inter-personal interactions with informal social network ties. People tend to acquire healthcare knowledge from others who share similar lived experiences of using healthcare services, which binds the knowledge seekers through homophily. Due to the social responsibility to help others being ingrained within the community, people (predominantly women), support each other, providing emotional and instrumental support in addition to essential healthcare information. This then builds holistic healthcare literacy, which people conventionally do not gain solely from the knowledge transmitted by healthcare professionals.

**Conclusion:**

People in diverse community settings acquired, co-constructed, transmitted, or suppressed knowledge about various healthcare services with the support of informal networks, mostly family and friends, combined with mass media sources. Therefore, people’s healthcare knowledge is not an individual asset but a shared resource among their social circles. It is multi-faceted and acquired from diverse sources available in the local and online communities and not limited only to individually held lay accounts of using healthcare services.

**Supplementary Information:**

The online version contains supplementary material available at 10.1186/s12913-025-12525-y.

## Introduction

Health literacy, which can be defined as a combination of socio-intellectual skills to acquire, process, and comprehend knowledge of different aspects of health, is known to inform people’s attitudes and behaviours towards health service utilisation [1–3]. Previous work has mainly focused on evaluating knowledge at an individual level - primarily assessing ‘functional literacy’, which concerns cognitive knowledge and skills. However, functional literacy constitutes only one facet of people’s health literacy [4, 5]. Others posit that health management-related knowledge is also influenced by people’s social network of families, friends, public systems and services (place) in communities [6, 7]. It is recognised that health literacy develops when people seek and share knowledge with others surrounding them - influencing each other’s knowledge as ‘*literacy or knowledge mediators*‘ [7]. In forging relationships with other members of the community, people get exposed to the lay experiences or accounts of others’ experiences of accessing health services [8–11]. So, people together co-create health literacy through shared knowledge distributed across their social relationships [4, 7, 8, 12]. Understanding people’s knowledge of health services entails a significance because the increasing inequality in people’s utilisation of healthcare services due to the uneven distribution of various intellectual, social and economic resources at individual, interpersonal and structural levels is a global public health challenge [13, 14]. Over- and under-utilisation of healthcare services negatively impacts health outcomes and burden healthcare systems, which needs to be addressed in order to use healthcare resources to the best effect [15].

Nevertheless, literature on the phenomenon of people’s distributed knowledge of healthcare systems and services, which embraces a social network dimension, emerging from people’s actions of knowledge-seeking and sharing about healthcare services outside healthcare settings, in local and virtual places and environments, is a relatively under-studied aspect of health literacy. This literature is also not well-integrated. This paper, therefore, provides an overview of the existing literature on people’s knowledge-seeking and sharing of healthcare systems and services across social networks in various communities. In this review, a ‘*community*’ refers to a group or network of lay people comprising families, friends, relatives and acquaintances connected amongst each other either locally or online through valuable relationships who share common assets like neighbourhood, culture, beliefs, norms, identity and history, virtual spaces or simulated environments and participate in different activities together [11, 16, 17] and health-service related information of people will be referred to as *healthcare knowledge* throughout the review.

The aims of this scoping review were to: (1) map the types of studies undertaken; (2) collate and summarise study population characteristics, theoretical frameworks, and critical findings from the included studies; and (3) identify potential gaps for further research. The research question was formulated according to the target population (local and online communities), the phenomenon of interest (people’s knowledge seeking and sharing across social networks) and study outcome (health service utilisation). The research question was therefore framed as follows: *How are knowledge and beliefs acquired*,* constructed and exchanged in different community settings*,* influencing people’s decision-making to help themselves*,* their families*,* and community members access healthcare services?* Supplementary Table 2 contains further detail regarding the research question formulation.

## Methods

### Study design

This study conducted a scoping review following Arksey and O’Malley’s [18] enhanced framework proposed by Levac et al 2010 [19]. The enhanced framework provided guidance on deciding the extent of the review’s exhaustiveness in the given time frame and available resources, thus providing more clarity to the authors about the practical challenges and limitations to be considered while implementing the methodology, which was further adapted as per this scoping review’s research question and aims. Supplementary Table 1 contains further detail regarding the process of the scoping review method undertaken.

### Literature search

An electronic search was developed using search terms from key articles identified from a preliminary search. The keywords were categorised into target population, phenomenon of interest and study outcome and combined to form a search strategy (Supplementary Table 3), which was then run across different databases, amending the search by incorporating relevant MeSH terms and subject headings and applying relevant proximity and Boolean operators (amended search strategy, Supplementary Material 1). Four databases (Ovid Medline, Web of Science, CINAHL, and PsycINFO) were searched. Search results were limited to English, and studies from the last 20 years were included in this review. Title-abstract screening was undertaken using the study eligibility criteria (Table 1). To make the title-abstract screening process methodologically rigorous, in addition a second reviewer independently reviewed 5% of abstracts and was blind to a first reviewer’s assessment. Reference management software (Rayyan.ai) was used to organise and catalogue decisions and compare the assessment of the two reviewers. Any discrepancies were discussed and resolved and helped to ensure that the criteria used could be applied objectively. The full text of the selected papers was then screened to identify studies to be included for data charting and reporting.

**Table 1:** Eligibility criteria to select relevant studies using the SPIDER framework

Inclusion criteria	Exclusion criteria
**Sample[S]**: Participants of all age groups from different generations meeting the criteria of local or online communities as defined above were included in the review	**Sample [S]**: Study samples not meeting the criteria of local or online communities as defined above were excluded from the review.
**Phenomenon of interest [PI]**: Studies were included if they have evaluated or explored the phenomenon of interest: (1). Healthcare-related knowledge and belief construction or exchange or informational or appraisal support within different community settings amongst families, friends, relatives and other community members through social interaction, shared experiences, storytelling, and narratives shaping decision-making; (2). Healthcare-related knowledge acquired by people through mass communication media: (a). Internet (b). electronic or (c). print and shared in their local or online communities.	**Phenomenon of Interest [PI]**: Studies that have not investigated the phenomenon of interest as mentioned under inclusion criteria were excluded. Furthermore, studies were excluded if they have evaluated or explored: (**1**). Healthcare-related knowledge practices and beliefs of healthcare professionals; (**2**). Evaluation of lay people’s healthcare-related knowledge and beliefs or healthcare literacy by professionals only in terms of their reading, writing or information processing skills using standardised questionnaires; (**3**). Studies which evaluated only instrumental support in communities (**4**). Studies on healthcare promotion and healthcare education (**5**). Studies on online communities that have not investigated knowledge and belief construction or exchange by people in local and online communities. Overall, any study conducted to evaluate lay people’s healthcare-related knowledge and beliefs from a professional viewpoint instead of the participants themselves were excluded.
**Design [D]**: The scoping review included all study designs. 1. Empirical studies (quantitative, qualitative, and mixed-method studies) 2. Literature reviews, all types, including narrative, systematic, with or without meta-analysis, overviews, realist reviews, critical interpretive reviews, and umbrella reviews. 3. Theoretical articles- on relevant sociological concepts and theoretical frameworks	**Design [D]**: Not applicable, as all study designs were included.
**Evaluation [E]**: Healthcare service utilisation	**Evaluation [E]**: Studies emphasising general health and well-being and not addressing healthcare service utilisation primarily were excluded.
**Research [R]**: All studies, including primary (quantitative, qualitative, mixed-method), all types of literature review and theoretical studies, and grey literature were included.	**Research [R]**: Conference abstracts, editorials, commentaries, and studies published in languages other than English were excluded

### Data charting and reporting process

Extracted data included the general characteristics of study populations, any relevant theoretical frameworks or emerging concepts if used; how authors have applied them and findings relevant to the topic of interest. Findings from quantitative studies were described narratively thus contextualising statistical data as the principal objective of this review was to map the nature and breadth of the evidence and integrate it. The critical findings concerning people’s healthcare-related knowledge were then categorised into type (intra- or inter-generational), actors, source and medium. In this review, the actors or mediators of healthcare knowledge referred to individuals engaged in health service-related information seeking or sharing, categorised as close (strong) or loose (weak) social network ties. Sources of healthcare knowledge refer to any person or object from which people gained information. Family members and friends were considered close ties, whereas relatives, coworkers, acquaintances, community members, healthcare professionals, community centres and organisations were regarded as loose network ties.

## Results

**Fig. 1:**
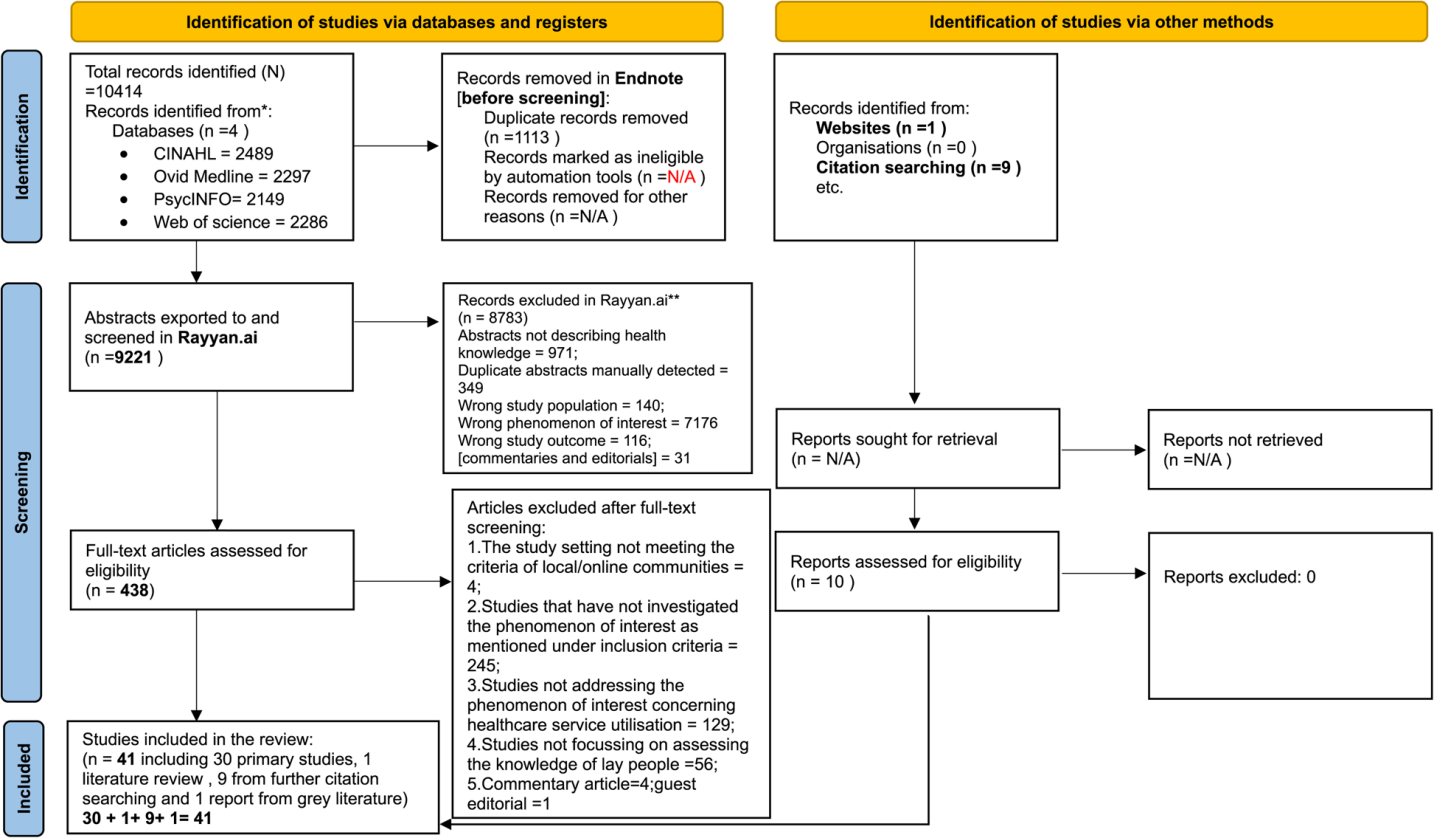
A schematic illustration of the steps performed during the screening process adapting the PRISMA 2020 flow diagram Automation tools have not been used during the screening to exclude studies (**). Source: Page MJ, et al. BMJ 2021;372:n71. 10.1136/bmj.n71

The search yielded 9221 abstracts reviewed by the first reviewer. The second reviewer independently reviewed 450 of these. The two reviewers’ decisions matched apart from seven disagreements, which were discussed and reduced to two disagreements, leaving zero after the full-text screening was undertaken. Out of 438 articles identified for full-text screening, 31 were identified for inclusion. Nine additional studies were identified through reference chaining. The grey literature search yielded one report, making a total of 41 included studies for data extraction (Fig. 1).

### Theoretical or conceptual frameworks used

A wide variety of theories and concepts were used and can be categorised into four overarching types (Table 2). Most of the empirical studies (*n* = 12) applied theories concerning knowledge, information or literacy; 8 studies used concepts relating to social network structure; 5 studies used different versions of the Social Ecological Model (SEM) model, while only 2 studies used the concepts of social and cultural capital respectively. Twelve studies did not cite any theory. Several authors adapted theories to construct a theoretical framework or to inform their study design and data analysis. In a few studies, some concepts were identified as a consequence of study findings, such as the concept of ‘diffusion of information’ [20]. The most relevant concept concerning people’s shared knowledge was *‘Community health literacy’*, which concerned people’s collective local knowledge and skills acquired by social interactions across generations apart from cognitive knowledge [21]. Theories/concepts identified are all detailed in Table 2.

**Table 2:** Theories used

No	Study citation	Theory used in the study [Name] or concept	Differentiation between health-related and healthcare-related knowledge? Yes/No (Is the author conceptualising health and healthcare knowledge distinctly)	Concepts recognised to be important concerning lay people’s healthcare-related knowledge practices* in various community settings
**Theories/concepts based on the Social Ecological Model (SEM):**				
**1.**	Shahabuddin et al. 2019 [22]	**Social Ecological Model (SEM)**: The society can be divided into four hierarchical levels; inter-connected with and influence each other: 1. Individual level 2. Family level 3. Community level 4. Health systems- Structural level	Yes	Women who lack optimal knowledge of maternal health services are surrounded by family, community who have experiential knowledge of using maternal services during pregnancy which helps them to make maternal healthcare-related decisions.
**2.**	Kawaguchi et al. 2021 [23]	**Social-ecological model of health**: The process of decision-making can be segregated into: ▪ Individual level ▪ Interpersonal level ▪ Community level ▪ Societal level	No	Healthcare decision-making process at interpersonal and community levels is done through interfamilial communication to select the healthcare facility for the delivery of the child.
**3.**	McEwan et al. 2014 [24]	**Social- Ecological Model [SEM]**: Two concepts grounded in SEM: 1. Kleinman’s theory of explanatory model 2. The health belief model	yes	Lay people create their own meanings about disease symptoms, diagnoses, and treatments, which inform their actions or advocacy for healthcare.
**4.**	Herrero-Arias and Diaz, 2021 [25]	**1. Socioecological concept**: Ungar’s socioecological concept **2. Social navigation concept**	Yes, (Addressed as “knowledge of healthcare services”).	Immigrants use their social networks to learn how to negotiate with healthcare providers in an unfamiliar healthcare system in a foreign country.
**5.**	Brennan-Ing et al. 2023 [26]	**Relationship between Health Locus of Control [HLoC] and social support**: The balance between internal and external factors controlling health- the locus of control.	No	Grandparents taking care of grandchildren are an integral source of health information.
**B. Theories based on social capital elements**:				
**6.**	Park et al. 2021 [27]	**Social capital theory**: Social resources as assets: (A) **Structural** social capital- Social network, (B) **Cognitive** social capital- Social cohesion and trust	Yes	Social networks contribute to people’s awareness about healthcare services.
**7.**	Madden 2015 [28]	**1. Cultural Health Capital (CHC)**: One of the resources people use to navigate the healthcare system. **2. Community Cultural Wealth (CWC)**: Resources harboured specifically by a marginalised group; comprised of six distinct components.	No, (US healthcare-related knowledge is called health knowledge)	The degree of dependence on social networks to obtain appropriate health services increases several-fold for some communities, who fear getting misjudged or stigmatised due to their unauthorised residency in a foreign country, where people are compelled to avoid visiting healthcare systems and are left with no other resources except obtaining informational support from close network ties.
**C. Theories/concepts based on social network formation and structure**:				
**8.**	Felsher et al. 2021 [29]	**Social network theory**: The egocentric analysis maps how an individual [**ego**] is connected to other people [**alters**] across the social network to understand the architecture of social circles and the influence of ‘alters’ on the behaviours of ‘egos’.	No	The ego-centric social network analysis facilitates identifying key actors engaged in healthcare knowledge sharing in social networks.
**9.**	Amoah et al., 2018 [30]	1. Social networks harbour different resources, such as social ties and knowledge. 2. Social network structure can be divided into three distinct levels: *Micro level* *Meso level* *Macro level* **3. Social Organisation Strategy [SOS] framewor**k Decision-making is not an individual but a collective mechanism of social interactions across the social network	No, (The author addressed healthcare access information as a part of health-related information)	Medical decision-making is a product of social interactions across social networks because social network ties provide informational support.
**10.**	Comfort et al. 2022 [31]	**Social Network Theory (SNT)**: People’s health behaviour is influenced by their social networks. These networks serve as a storehouse of various resources, such as informational, appraisal, instrumental, and emotional social support, facilitating or restricting healthcare-seeking.	Yes	Informational support offered by various social ties—strong and weak—influences women’s decision-making about seeking antenatal care services.
**11.**	Konstantinou et al. 2021 [32]	**1. Social Network Theory (SNT)**: Interpersonal relationships in society. 1. **Egocentric network**– Egos and alters 2. **Sociocentric network**– Interpersonal communication **2. Social contagion theory**: A person’s perceptions are transmitted to others, influencing their behaviour.	NR	An individual’s knowledge and beliefs about immunisation healthcare services are transmitted to another spreading across the social network impacts people’s behaviour of accepting and utilising services. Highlights concepts in the social transmission like centrality, clustering including homophily.
**12.**	Goldberg 2014 [33]	**Theories on social networks and social norms**: 1. Social networks and social capital are related. Social capital is embedded within social relationships. 2. People’s actions are the results of their social networks. 3. Social networks possess injunctive and descriptive social norms.	Not mentioned explicitly	The healthcare knowledge mothers gain by communicating with their social network ties, combined with social norms, shapes their decision-making capacity, which determines their children’s immunisation uptake.
**13.**	Murakami et al. 2019 [34]	Social relationships comprised of social networks where social support play a crucial role in diffusing information and influencing behaviour.	Yes	Diffusion of information across social networks influences people’s dental service utilisation.
**14.**	Schafer 2013 [35]	Healthcare utilisation is encouraged by social network ties. This conceptual framework used the following theories: **1. Andersen’s behavioural model** **2. Pescosolido’s Social Organization Strategy (SOS)** and **3. Network Episode Model (NEM)**	Yes	People’s social networks provide different types of social support, including informational and appraisal support. People’s discussion networks influence the utilisation of different types of healthcare services.
**15.**	Kettle et al. 2019 [36]	**Family practices**	No, (Professional dental healthcare has been described as dental health)	Experiential knowledge of family members builds family practices of healthcare visits, which can change across generations with time.
**D. Theories/concepts based on dynamics of knowledge/information/literacy at the interpersonal level**:				
**16.**	Hill et al. 2018 [37]	**Cohen and McKay’s classification of social support** Cohen and McKay’s (1984) concept of social support. Social support derived from formal and informal social networks– Instrumental support, emotional support, appraisal support and informational support.	No	The healthcare-related information is sought or provided by an individual to another: 1. **Informational support** is the sharing of knowledge to resolve any problem. 2. **Appraisal support**, where an individual is exposed to healthcare information from others around them actively or passively.
**17.**	Amrita and Roy, 2019 [38]	**Knowledge transfer** **Knowledge sharing** and **diffusion** in rural communities through various sources for assistance in decision-making.	Yes	The interpersonal healthcare-related knowledge transfer is the human behaviour of inducing information from one individual to another in a social network.
**18.**	Warren-Jeanpiere et al. 2010 [39]	Inter-generational gynaecological healthcare communication	Yes	Inter-generational communication of mothers with their daughters about gynaecological visits
**19.**	(A). Bradby et al. 2019; [40] (B). Phillimore et al. 2019 [41]	**1. Healthcare bricolage**: People in a neighbourhood use locally available resources,, including knowledge and network ties, **2. Role of the super-diverse neighbourhood in accessing health services**: A super diverse neighbourhood shapes people’s access to health services.	Yes (Described as healthcare communication)	In super diverse neighbourhoods, knowledge is one of the resources residents leverage when they need to access healthcare services.
**20.**	Samerski 2019 [42]	**Health literacy as a social practice** “Health literacy as social practice” integrates mainly three theories: 1. Practice theory 2. New literacy studies- Education as a ‘**sociocultural practice**’. 3. Praxeological theory	Yes, (Health and healthcare is described separately but predominantly uses health knowledge)	Literacy is a socio-cultural practice and people co-produce healthcare knowledge with others.
**21.**	Foster et al. 2016 [43]	**Lay health-related knowledge** Two forms of lay health-related knowledge: 1. Explicit knowledge informs scientific facts and evidence 2. **Tacit health knowledge** emerges from social skills, thoughts and life experiences	The author differentiated between two forms of lay health-related knowledge: explicit knowledge and tacit knowledge.	Tacit healthcare knowledge is co-constructed and exchanged among people in a group to deal with their illness
**22.**	Ward et al. 2021 [44]	**1. Informational support interspersed in social networks** **2. Information-motivation-behavioural skills model** Health behaviour changes due to three major factors: information about the behaviour, motivation to change the behaviour, and social skills people apply to change the behaviour.	Yes	People provide informational support to experientially similar friends affected by the same condition through medical treatment-related advice or recommendations
**23.**	Threats 2020 [45]	**Wilson’s model of information behaviour framework [1981]** Information-seeking behaviour is divided into [1] Information seeking: active-Interaction with others and receiving passive information from others but not using it [2] Information utilisation	No	To address the health and healthcare information needs, people seek different sources to construct their information, consume the information and transfer it to others, who will further exchange it with others through various sources
**24.**	Celentano et al. 2021 [20]	**Diffusion of information** Information is diffused from one person to another.	No	Healthcare-related information diffuses from school to home through children to mothers.
**25.**	Metcalf et al. 2013 [46]	**1. System dynamics** The system where people’s behaviour influences each other in a complex system such as an organisation or ecosystem. **2. The classic Bass model of S-shaped diffusion over time (1969)** Diffusion of healthcare-related information via people’s interactions.	No	Word of mouth can be described as a phenomenon in which people seek healthcare-related information in their social circle, receive it from people using healthcare services, and spread it to others.
**26.**	Tatari et al. 2021 [47]	**Ripple effect**: The passing-it-on approach or snowballing of healthcare information to another person	yes	The desire of several women to share cancer screening-related knowledge gained by them during a health promotion event, with other women in the community
**27.**	WHO 2022 (Grey literature) [21]	**Community health literac**y: The sum of individual health literacy, which every person gains through social interactions across generations, health customs originating from social norms, and everyday life practices.	No, Health literacy used as the umbrella term encompassing health-service-related knowledge.	The concept of community health literacy could be translated to understand the shared knowledge possessed by people in a community about different healthcare services.

*For this review, people’s healthcare-related knowledge practices in different types of communities refers to the knowledge which develops when people take action to search for or share healthcare-service-related information across their social networks leveraging various formal, informal, and materialistic sources at interpersonal and community levels

### General characteristics of included studies

Studies were identified from 15 countries across North America, Europe, Africa, and Asia and were well distributed in urban, rural, and virtual communities. Most studies (*n* = 17) were conducted in the USA, followed by 4 UK studies. More studies (*n* = 29) have been conducted in high and upper-middle-income countries compared to lower-middle and low-income countries (*n* = 9) [48]. Most studies were conducted in local areas or neighbourhoods (*n* = 27). Twelve were set in urban and suburban areas, and 4 were in rural ones, with another 3 involving both rural and urban settings. Instead of a particular place, several studies (*n* = 9) focused on a specific group of people sharing common characteristics such as gender, immigration status, ethnic minority status, state of deprivation, or any health condition living in a particular district, state or country, while 3 studies were conducted exclusively on online communities (2 private groups in USA, 1 global) inter-connecting people across different states and countries which transcended local residence boundaries (Table 3).

**Table 3:** General characteristics of included studies

No.	Citation Author and publication date	Study setting, Country	Nature of health service utilised	Type of study	Type of community setting [Rural/suburban/urban/Any specific group/online]	Socio-demographic characteristics of the study population			
						Age Mean/Range	Gender distribution	Ethnicity	Socio-economic conditions, including education
**General healthcare services**:									
1	Amoah et al. 2018 [30]	Ghana, Africa	General healthcare	Qualitative	**Rural and urban settings**: 36 urban and 8 rural communities in the Ashanti region of Ghana	Range: 15 years- >60 years Most of the participants were aged 25–44 years	Women = 53% Men = 47%	Specific ethnicity not reported	Education status: Junior high school for most of the participants
2	Phillimore et al. 2019 [41]	**Multi-centre**,** multi-national**: Birmingham, Bremen, Lisbon and Uppsala in the UK, Germany, Portugal and Sweden [Europe]	General healthcare	Qualitative	**Urban settings**: Two superdiverse neighbourhoods each in four European cities.	Range: 27–33 years	Women = 1 Man = 1	Immigrants in European countries, including one asylum seeker, (Specific ethnicity not reported)	NR
3	Bradby et al. 2019; [40]	**Multi-centre**,** multi-national**: UK, Germany, Portugal, Sweden [Europe]	General healthcare	Mixed-method: 1.Qualitative 2.Quantitative	**Urban settings**: Two superdiverse neighbourhoods in one city of each country-Birmingham Bremen Lisbon Uppsala	Range: 18 years- > 80 years	Women = 985 Men = 770	Multicultural- Specific ethnicity not reported	Education status: Approximately one-third of the female (35.9%) and male participants (36.1%) had higher education. Occupation status: NR Income status: Participants’ income ranged from very low to high, with the majority under lower income.
4	Samerski 2019 [42]	Bremen, Germany	General healthcare	Qualitative	**Urban settings**: Super diverse neighbourhood settings, local community centres, local Ambulance and Emergency centres [A and E]	27–87 years	Women = 22 Men = 20	Multicultural [specific ethnicity of individual participants has not been reported]	NR (Many participants were first and second-generation migrants)
5	Foster et al. 2016 [43]	Virtual	General Healthcare	Qualitative	**Online community**: “HealingWell” has 150,000 registered users. It is part of a general health website comprising 32 English-speaking forums for different illnesses, a chatting room, a blogging section, newsletters, and an e-book library that provides informational resources to users.	NR [As this was an online community]	NR	NR	NR
6	Herrero-Arias and Diaz, 2021 [25]	Norway, Europe	Norwegian public, primary and secondary healthcare services	Qualitative	**Urban settings**: Three municipalities in Norway which have Southern European immigrant residents	30–50 years	Women = 15 Men = 5	Southern European: Spain = 15 Greece= Italy = 2 Portugal = 1	Education status: University = 14 Secondary = 4 Primary = 2 Occupation status: Employed = 19 Unemployed = 1 Duration of residency in Norway [in years]: 10–15 = 2 5–10 = 11 1–5 = 7
7	Izquierdo et al. 2018 [49]	California USA	VA (Veteran Affairs) health services (VA care) for veterans- provided by Veterans Health Administration (VHA)	Qualitative	**Urban setting**: A deprived region in South Los Angeles County where veterans live		Men = 10 Women = 5 (2 veterans, 3 family members)	African-American	Lack of Veteran Affairs (VA) healthcare facilities within the vicinity.
8	Schafer 2013 [35]	USA	Physician visits, Complementary and Alternative Medicine (CAM) treatment	Quantitative	**Setting**: Not reported	57–85 years	Gender distribution was not reported; male and female participants were included.	Hispanic, Black	Not reported
**Viral disease-related healthcare**,** including preventive services**:									
9	Hill et al. (2018) [37]	USA	HIV/AIDS-related healthcare services	Qualitative	**Rural**: South-eastern health districts comprising of rural areas	20–60 years	Women-9, Men-11, Transgender-1 Sexual orientation: Heterosexual- 10 Homosexual– 7 Bisexual- 1 Not sure- 3	1. African American = 12; 2. White (non-Hispanic) = 6; 3.Native American = 1, 4. Not reported = 2	NR
10	Felsher et al. 2021 [29]	Philadelphia, USA	HIV preventive care: Pre-exposure prophylaxis	Qualitative study,	**Specific group**: Women who inject drugs enrolled in a community programme on syringe services.	≥ 18 years old, Mean age = 39 years	Women who inject drugs	1. Non-Hispanic White-14 2. Non-Hispanic Black-4 3.Others-2	Education status: NR Housing status: Homeless = 12 Income status: <$5000 = 10 Health insurance coverage = 18 Self-reported poor health status = 15
11	Whitford et al. 2021 [50]	Indonesia, Asia	1. HIV testing [predominantly] 2. Sexually Transmitted Infection [STI] testing	Qualitative	**Urban settings**: Residents of three cities, Bandung, Denpasar, and Yogyakarta, who lived in brothels or worked in bars or nightclubs	≥ 16 years old	Women	Specific ethnicity not reported	Education status: NR Occupation status: Direct or indirect sex worker 3. HIV status: HIV-positive without treatment = 16 HIV-positive receiving treatment = 18 Undisclosed status = 27
12	Threats 2020 [45]	North Carolina, USA	1. HIV care	Mixed-method	**Urban**,** suburban and rural settings**: 1.Urban districts = 64% 2. Sub-urban disctricts = 14.4% 3. Rural counties = 21.6% Young Bisexual and Gay Men (YBGM) residing in different counties of North Carolina	Mean = 29.2 years	Men = 78 Transgender = 3 Two-spirit (third gender) = 1 Not reported = 1 **Sexual** **Orientation**: Homosexual = 67 Bisexual = 13 Other = 3	Black men who report themselves as gay or bisexual.	Education status: 1.55.42% did not have a graduation Employment status: 74.7% were full/part-time educated Income status: <$20,000–34% $20,000-$39,000 = 36% Health insurance coverage status: Insured = 67.5% Uninsured = 32.5%
13	Ward et al. 2021 [44]	Baltimore, Maryland, USA	Hepatitis C Virus [HCV] healthcare targeted for People Who Inject Drugs [PWID]	Qualitative	**Urban setting**: HCV + ve people who inject drugs in Baltimore city.	32–65 years	Women = 5 Men = 15	African American = 15 White = 5	Education level: NR* Occupation status: Employed (full/part-time) = 3 Unemployed= 17 Income status: <$1000/month = 15 Health insurance coverage status: (Medicaid) 17 Housing status: Homeless = 10
14	Mascia et al. 2020 [51]	Italy	Paediatric immunisation services	Quantitative	**Specific group**: Students in a school	NR	45% girls 55% Boys	Italian- 45 (92%) Other ethnicities = 4(8%)	Education status: Class 1 = 37% Class2 = 25% Class3 = 18% Class 4 = 20% Income status: Not applicable to school students
15	Goldberg et al. 2014 [33]	Bungudu, Nigeria	Pediatric immunisation services	Quantitative	**Rural setting**: Villages under a local government region are grouped as low- and high-immunisation villages.	25–34 years	97% women (Mothers), 3% caregivers	Hausa- 539 Kanuri-1 Fulani-10	Education: Most women were educated at Qu’ranic schools. Income status: Low quintile = 44% Middle quintile = 55%
16	Buller et al. 2019 [52]	USA	HPV vaccination	Quantitative	**Online**: Facebook private groups across 34 US states- online social networking platform	Mean age = 43.13 years	Women [mother]	White-86.6% Non-White-12.9% Prefer not to disclose-0.5% Non-Hispanic-92.8% Hispanic-6.5% Prefer not to disclose-0.7%	NR
17	Celentano et al. 2021 [20]	USA	HPV vaccination	Qualitative	**Specific group**: Immigrant mothers to the USA	Mean age of mothers: 41 years Mean age of adolescents: 15.1 years	Mothers of adolescent [11–17 years] children who are eligible for HPV vaccination	East African speaking either Somali, Amharic or Tigrinya	Education status: Mean years of formal education = 9.5 years Income status: Annual income ranges from <$25,000->$50,000, with the largely less than $25,000
18	Fu et al. 2019 [53]	Washington DC, USA	HPV vaccination	Quantitative	**Specific group**: Parents of children (10–12 years age) eligible for HPV vaccination	Median-37 years	94.1% Women	African-American	Education status: College = 41.9% High school = 45.3%
19	Casillas et al. 2011 [54]	Los Angeles (LA), USA	HPV vaccination	Quantitative	**Specific group in the urban county**: Women responsible for decision-making of their daughter or other girls’ HPV vaccination and contacting LA, office of women health	Mean age: 43.9 years	Women = 294	Latina = 55% Chinese = 22% Korean = 9% Afro-American = 9% Multiracial = 4%	Education status: High school = 40.2% College = 28.1% Income status: <$1000 per month = 34.2% Low income Health insurance coverage status: 1.Women respondants = 27.9% had insurance 2. Daughter or another girl Who needed HPV vaccination = 78.8% had insurance.
20	Lee et al. 2021 [55]	USA	HPV vaccination-preventive care for cervical cancer	Quantitative	**Specific group**: People of one of the ethnic minorities (Khmer community) living in the USA	Mean age: Mothers = 44.3 years Daughters = 15.3 years	1. Mothers 2. Daughters who are to start the HPV vaccination	Cambodian American	Education status: Mother-High school or college = 84.2% Daughter-High school = 84.2% Occupation [employment] status: Mother = 63.2% Daughters = 15.8% Income (per month) status [range]: Mothers: >$1000 = 4 (21.1%) to >$3000 = 6 (31.6%)
21	Ruiz 2015 [56]	Northern California, USA	HPV Vaccination	Quantitative	**Specific group**: Students of Northern California University	20.22 years (Young adults)	66.2% girls	Asian or Pacific islander (46%)	Education status: Senior students: 40% Junior students: 39%
22	Hernandez et al. 2019 [57]	USA	H1N1 vaccination	Quantitative	**Specific group**: Pregnant women with first child	Mean age: 29.9 years	Gestational women	Non-Hispanic white = 67.6% Non-Hispanic Black = 5.4% Non-Hispanic other = 21.5% Hispanic = 5.8%	Education status: Bachelor’s level: 38.8% Master’s level = 24.1% Doctoral degree = 9.8% Prof.degree = 5.8%
23	Brennan-Ing et al. 2023 [26]	New York, USA	Covid-19 vaccination	Qualitative	**Urban settings**: People from different linguistic-cultural backgrounds living in New York City.	65–94 years	Predominantly women, with a few men	US-black = 22, US- white = 8, Caribbean = 6, Chinese = 9, Hispanic = 21, Russian = 11	Income status: Low-income Education status: Some participants were college graduates
**Maternal healthcare services**:									
24	Gupta et al. (2015) [58]	Ghana, Africa	Maternal and child healthcare services, including prenatal, perinatal and postnatal care	Qualitative	**Rural**: Kassena-Nankana districts	NR	NR	NR	NR
25	Amrita and Roy, 2019 [38]	West Bengal, India	Maternity care: 1. The choice of surgical procedure for the delivery treatment; 2.Antenatal care	Quantitative	**Rural settings**: Two rural districts	Range= </=18–30 years; Mean age of the participants in two districts = 23.9 and 24 years	Women	Asian-Indian	Education status: No education to Masters Family members of the participants (range = 3–13 members) Income status: Low-income families
26	Comfort et al. 2022 [31]	Uganda, Sub-Saharan Africa	Antenatal care- Maternal care services	Qualitative	**Specific group**: Pregnant women at different stages of gestation in Uganda (Women’s localities not mentioned*)	18 years - ≥31 years	Women	Ugandan (Ethnic origin not reported)	Education status: Most women had secondary school education, Stage of gestation: Most women sought ANC care during the second trimester
27	Shahabuddin et al. 2019 [22]	Banke, Nepal	Maternal healthcare services-Antenatal and postnatal care for adolescents	Qualitative study	**Specific group**: A district selected which has the highest rate of adolescent pregnancy	14–19 years	Adolescent girls	The ethnicity of the participants was not reported. (The ethnic groups in the district included Tharu, Muslim, Chhetri and Brahman).	Education status: Predominantly secondary school education, some girls have no formal education with
28	Warren-Jeanpiere et al. 2010 [39]	USA, In 2004	Maternal healthcare: Gynaecology	Qualitative study	**Urban settings**: Community leisure centre and public university campus	20–55 years [mean age-31 years]	Women	African American	Education and employment status were varied. Health insurance coverage status: 13 participants
29	Kawaguchi et al. 2021 [23]	Laos, Asia	Maternal healthcare: Childbirth services	Qualitative	**Rural**: Eight villages in Xepon district in the Northeast Savannakhet province, Central Lao	17 years-38 years	Women who gave birth within one year of the study	Tri = 8 Mangkong = 4 Phoutai = 1 Laolung = 2 Vietnamese = 1	Education status: 9/16 without education Duration of education 0.9 years to 4.1 years Occupation status: Farmers Day labourers
**Oncology healthcare services**:									
30	Woof et al. 2020 [59]	England, UK	Breast cancer screening	Qualitative-cross sectional	**Specific group**: A neighbourhood with a predominant population of British-Pakistani in East Lancashire	> 50 years = 12 < 50 years = 2 NR = 2	Women who require preventive breast screening	British-Pakistani	Deprivation status [IMD index]: The study setting is considered under 10% of the most deprived in the UK Education status: Many women face difficulty in communicating, reading, and writing in English. Sociocultural linguistic differences.
31	McEwan et al. 2014 [24]	Egypt, Africa	Breast cancer diagnosis and treatment	Qualitative	**Specific group**: Women with delayed diagnosis of breast cancer from different places-Cairo, Alexandria and Delta	29–60 years	Women (All mothers)	Not reported explicitly	Education status: University degree = 4 High school = 5 Reading-writing skill = 4 Illiterate = 2
32	Tatari et al. 2021 [47]	Aarhus, Denmark	Cancer screening programme	Qualitative	**A specific group in a suburban setting**: Ethnic minority immigrants from non-Western countries living in socially deprived suburban settings in Gellerup in the Aarhus city of Denmark	27–59 years [median:39 years]	Immigrant ethnic minority women	Ten non-western countries, including Somali, Arabian and South-East Asian	Education status: Currently registered under educational institute: 5/37 Employment status: Employed including self-employed = 13/37 Unemployed = 13/37 Not reported = 6 Deprivation status: Gellerup comprised 6000 citizens, 81.3% of whom are immigrants, and is considered socially deprived, with 54% of the population unemployed.
33	Hwang et al. 2012 [60]	USA	Colorectal Cancer Screening (CRC)	Quantitative	**Online**: An online weight loss community group-SparkPeople.com	≥ 50 years Mean = 57.9 years	Women = 92.2%	Race: White = 92.7% Black = 4.4% Others = 2.9% Ethnicity: Hispanic = 2.2%	Occupation status: Employed full time = 50% Employed part-time = 13.2% Unemployed = 36.7% Education status: Bachelor’s or higher degree = 49.5% Health insurance coverage status: 93.4%
**Dental (Oral) healthcare services**:									
34	Murakami et al. 2019 [34]	Japan, Asia	Dental (Oral) Preventive and curative services During 2010–2011	Quantitative	**Urban settings**: Four municipalities are located in the Greater Tokyo region.	37.3 years	Women = 2207 Men = 1919	Japanese	Education status: University level = 43.5% College = 33.6% School level = 22.9% Occupation status: Not reported Income status: Mean income (in Japanese yen per year) = 3611.4 thousand ($25,000 approx.)
35	Metcalf et al. 2013 [46]	New York, USA	Dental health screening services	Quantitative	**Urban settings**: Older adults living in deprived residences in the urban settings of northern Manhattan in New York	NR Older adults	NR	NR	NR
36	Kettle et al. 2019 [36]	Edinburgh and Sheffield, UK	Dental services	Qualitative	**Urban settings**: Geriatric people living in two cities, Edinburgh and Sheffield	65–91 years	Men = 15 Women = 28	White British	Varied status of education and occupation (not clearly mentioned)
**Other healthcare services**:									
37	Park et al. 2021 [27]	USA	Alzheimer’s disease health services	Quantitative	**Urban settings**: Older Korean Americans (ethnic minority) living in five different capital cities across five USA states-Los Angeles, New York City, Austin, Honolulu and Tampa	Mean: 73.41	Men= 33.2% Women = 66.8%	Korean- American	Education status: School level = 60.1% College and higher = 39.7%
38.	Madden 2015 [28]	USA-Mexico border	Trans-border healthcare services-Pharmacy (prescription drugs)	Qualitative	**Deprived settings**: Deprived communities in South Texas counties of Cameroon and Fidalgo at the USA-Mexico borders	18–73 years 80% between 30–60 years	Women = 26 Men = 18	Mexican/Mexican-American or Hispanic = 39 Non-Hispanic Whites = 4 Native American = 1	Occupation status: Self-employed = 4 Retired = 3 Unemployed = 3 Government benefits = 10 Students = 5 *Most participants belong to the working class and earn moderate-to-low hourly wages.* Health insurance coverage status: Without health insurance = 19
39	Townsend et al. 2014 [61]	British Columbia, Canada	Rheumatoid arthritis (RA) diagnosis	Qualitative	**Specific gender in rural and urban settings**: Women living in both rural and urban settings in northern and eastern British Columbia	30–70 years	Women seeking RA diagnosis	NR	Occupation status: Employed either full-time, part-time, retired or on sabbatical

Participants ranged from 14 to 94 years, comprising adolescent girls [22] to older populations like grandmothers [58], although 30–60 years of age was the most prevalent age group. Studies included women, men, and transgender people, with 19 studies focused exclusively on women, including 5 studies on mothers. Two studies [37, 45] conducted on HIV-affected people included transgender people and recorded the sexual orientation of participants. Predominant ethnicities studied were White and Black (African-American), followed by Hispanics and non-Hispanics, while several studies were conducted with immigrants. Most studies recorded the participants’ education and occupation, with a few studies recording the income status, deprivation index, health insurance coverage status, and self-reported health. Several studies involved low-income participants, and some were conducted in socially deprived communities. Six studies lacked data relating to either the age group, gender, ethnicity, or socioeconomic status of the study population.

### Types of study conducted

Most studies were qualitative (*n* = 23), followed by quantitative (*n* = 14) and mixed-methods (*n* = 2) (Table 3). Sixteen of the quantitative studies, including mixed-method studies, used questionnaires, where participants’ responses were recorded using pre-constructed instruments to quantify general people’s social networks and their knowledge and awareness about different health services to evaluate the association between these two variables. Four studies conducted Social Network Analysis to identify people (nodes/egos) involved in knowledge transmission, of which only one study [38] assessed degree centrality to find the principal source of knowledge transfer. One study [52] conducted an interventional Randomised Controlled Trial (RCT), while another study [46] conducted a system dynamics study using a simulation modelling technique.

Of the 25 studies, including mixed-methods studies using qualitative methods, 23 involved semi-structured interviews (11 exclusively; 12 in combination with other data collection methods, out of which 4 studies conducted ethnography, including 3 overt participant observations and one covert netnography). While seven studies combined focus groups with interviews, only 1 study [20] used focus groups as the single method; 1 study [49] conducted engagement workshops, and 1 study employed longitudinal two-phased interviews [22] in contrast to cross-sectional interviews.

### People’s knowledge-seeking or sharing about different health services in various community settings

The included studies examined people’s network ties, thoughts, intentions, and attitudes when communicating about different health services. All quantitative and qualitative studies showed that people in different countries and community settings, amid different circumstances, turn to informal social network ties to interact with their family, friends, and community members and use various information broadcasting resources to seek, support, share and make decisions about accessing different healthcare services. These ranged from general, preventive to specialised healthcare services, including for sexually transmitted, oncological, maternal, chronic degenerative and dental diseases. In contrast, one study [34] found that informational support from social circles was not associated with dental service utilisation. The findings showed that through informal social interactions, people conveyed their lived experiences, beliefs, and perceptions of healthcare services with other people for all types of healthcare services investigated in the included studies. Disagreements with healthcare professionals, lack of trust in physicians, immigration to a foreign country or a place with an unfamiliar healthcare system, and failure to access public healthcare systems as unauthorised immigrants [28, 40, 42] were some critical circumstances that compelled people to look for healthcare-related information from informal sources. Key findings of every quantitative and qualitative study are presented in detail in Tables 4 and 5, respectively.

**Table 4:** Quantitative studies

No.	Citation	Type of study design	Sample size	Type of healthcare knowledge searched or shared social networks [inherited, tacit, experiential, experientially similar, scientific/objectivist, praxeological]	Type of actors engaged in knowledge sharing (This section describes who is involved in information dissemination in the social circle: strong ties or loose ties– family, friends/peers, relatives, community)	Medium and source of knowledge sharing (beliefs, culture, norms, daily life practices], inherited, tacit, experiential, lay, experientially similar, scientific/objectivist, praxeological)	Variables measured to evaluate knowledge sharing and its outcomes	Key findings (The Influence of healthcare knowledge practices on people’s healthcare outcomes: Decision-making, healthcare visits, healthcare behaviour)
1	Amrita and Roy, 2019 [38]	Social Network Analysis (SNT)	306	1. Intergenerational and 2. Intragenerational	**Closed ties**: husbands, mothers-in-law, parents, friends, **Loose ties**: Relatives and healthcare professionals **Central actor**: The social network analysis showed husbands as the most consulted person.	**Source**: Social ties **Medium**: Social interaction with network ties	**Nodes** depicting the social ties/sources of knowledge **Degree centrality** to determine the central actor in the knowledge network	The nodes and edges in the social network analysis showed that rural women acquired maternal care service-related knowledge from formal and informal network ties, including family, friends and healthcare professionals.
2	Buller et al. 2019 [52]	Randomised controlled trial	*N* = 881	Intergenerational: Experiential, including experientially similar	**Close ties**: Mothers and daughters	**Source**: Experientially similar mothers **Medium**: Online interaction	Mothers’ degree of engagement with HPV vaccination posts is evaluated by the total number of reactions and comments to each post.	Mothers interacted on Facebook with other mothers to share their thoughts and experiences in making the decision to get their daughters HPV vaccinated. Some comments on posts unravelled contradictory perceptions of mothers about their children’s vaccinations.
3	Hwang et al. 2021 [60]	Questionnaire survey	*N* = 2386	Intragenerational- Experiential	**Close ties**: Friends Central actor: NR	**Source**: Friends **Medium**: Narratives of people’s personal experiences of Colorectal Cancer Screening (CRC)	Attitudes to CRC were evaluated using six social influence items: The social influence of family and healthcare professionals in CRC screening. The intention to share/receive CRC narratives from other members was measured.	Among 1618 participants who had lived experiences of CRC screening, around 40% of participants reported wanting to share or learn about CRC screening from other participants.
4	Threats 2020- Quantitative Component [45]	Questionnaire survey	*N* = 83	1. Intergenerational 2. Intragenerational: Experiential, scientific(objective) and praxeological knowledge-Intragenerational	**Closed ties**: Family members-partners, friends **Loose ties**: Doctors colleagues, casual sex partners	**Source**: 1. Close and loose social ties 2. Internet- Social media, search engines, government websites, Newspapers, Brochures, Television **Mode**: Social interaction and passive learning from various media sources.	1. HIV-related Information seeking behaviour items evaluated: (a). Sources of HIV-related information (b). Type of information I. Association of trust with the information sources Motivators and demotivators of HIV screening	Participants sought HIV screening and treatment-related information from wide-ranging sources- People in the participants’ social circle, including healthcare professionals and various media sources, motivated them to take pre-exposure prophylaxis.
5	Park et al. 2021 [27]	Cross-sectional (Survey using a standardised Questionnaire)	*N* = 2150	Not reported	**Closed ties**: Family, Friends	**Source**: Close ties **Mode**: Personal experiences and beliefs (Perceived knowledge)	Two dependent variables 1. Perceived knowledge about Alzheimer’s disease (AD) 2. Awareness of AD-related services Social network assessed by: 1. Number of family members/friends interacted at least once in a month 2. Confidential Interaction with family/friend 3. Closeness with family/friends	Alzheimer’s Disease (AD)-related knowledge and awareness of AD-related health services were positively correlated to social network ties- family, particularly married partners (*r* = 0.16, *p* < 0.001), friends with lived experiences of AD, and community participation.
6	Goldberg 2014 [33]	Cross-sectional Social network analysis (Ego-centric)	*N* = 550	Intragenerational- Experientially similar	**Closed ties**: Mothers Peer networks **Loose ties** Opinion leaders	**Source**: Social network ties **Mode**: 1. Communication with peers and opinion leaders. 2. Social norms around vaccination instilled in mothers	1. Social networks of mothers 2. Social norms (injunctive and descriptive) around immunisations for children 3. Immunisation perceptions of the social networks of mothers	1. Participants preferred to connect with experientially similar friends, and the social norms of peers and opinion leaders about children’s immunisations influenced mothers’ decision-making. 2. The rate of communication among participants and network ties influence social norms around immunisations.
7	Lee et al. 2021 [55]	Survey using a standardised Questionnaire	*N* = 38 Mothers = 19 Daughter = 19	Intergenerational- Experiential	**Close ties**: Mother-daughter **Central actor**: Not applicable	**Source**: Close ties **Medium**: 1. Dyadic mother-daughter healthcare communication with mutual trust in each other 2.The social norm of immunisation to prevent a disease	1. HPV vaccination intention 2. Mother-daughter health communication 3. Perceptions of Mother and daughter for each other’s vaccination-related decision-making.	Most mothers (84.3%) and daughters (78.9%) reported that they communicated about HPV vaccination freely with each other, with a few pairs restricted due to cultural, language, and emotional barriers.
8	Fu et al. 2019 [53]	Cross-sectional survey	*N* = 353	Intragenerational- Experientially similar.	**Closed ties**: Spouse-29.2% Family-87% Friends-45.6%	**Source**: Social ties **Medium**: Advice from trusted social contacts	1. Trusting family, friends and near ones for child’s HPV-vaccination-related advice 2. Social networks of parents providing HPV-vaccination-related advice 3. Network density- The number of contacts in the social networks who knew each other. 4. The proportion of participants’ network ties possessing HPV vaccination perceptions.	1. **Homophily**- Parents found to trust other parents going through similar experiences of HPV-vaccination decision-making. 2. **Advice networks**: Parents had HPV vaccine-related advice networks, which were mostly family-centric. Two to three social contacts provided suggestions to the parents about whether their children should be vaccinated. 3. More than 60% of the participants presented negative understanding towards vaccination due to the reluctant behaviour of their family and friends towards HPV vaccination.
9	Casillas et al. 2011 [54]	Cross-sectional study using a telephone survey	*N* = 294	1.Intergenerational -Family 2.Intragenerational-Friends 3. Praxeological knowledge from objects.	**Close ties**: Family, friends **Loose ties**: Healthcare professionals	**Source**: 1. Social ties, and 2. Mass media, including TV commercials, news, newspapers, radio, and the internet.	1. Social source of HPV vaccination-related information 2. Social discussion of HPV vaccination-related information, 3. Perceived HPV vaccine effectiveness	Women who learned about HPV- vaccination with family, friends, healthcare professionals, and mass media information broadcasting sources and discussed with social networks were more likely to perceive HPV vaccination as effective in comparison to participants lacking information sources and did not engage in HPV vaccination-related discussions.
10	Mascia et al. 2020 [51]	Cross-sectional questionnaire survey Social network analysis	*N* = 49	Intragenerational	**Closed ties**: Friends	**Source**: Network ties with friends **Medium**: Not reported clearly.	1. Social network of school friends 2. Social network of participants post-school	Friends influenced each other about immunisation and reported similar immunisation uptake.
11	Ruiz 2015 [56]	Cross-sectional (web-based survey)	*N* = 346 Vaccinated-163 Non-vaccinated-183	Intergenerational: 1.Experiential Knowledge 2. Praxeological knowledge	**Close ties**: Family **Loose ties**: Healthcare providers	**Source**: 1. Close and loose ties. 2. Internet **Medium**: 1. Communication with trusted network ties. 2. Digital information through devices. 3. Trust	1. Health-seeking behaviour 2. HPV vaccine decision making 3. HPV and HPV vaccination knowledge 4. Social network variables-Homophily and network density 5. Adoption of HPV vaccination across participants’ social networks	1. Increased utilisation of vaccination services was found to be associated with students whose family members had been vaccinated and were involved in the decision-making process. 2. Participants reached out to sources they trusted to acquire HPV-vaccination-related information. 3. Trust can be considered as a catalyst in the process of information seeking.
12	Hernandez et al. 2019 [57]	Cross-sectional study: Survey interview and questionnaire Social network analysis	*N* = 225	Not reported	**Closed ties**: Collegemates/peers	**Source**: Educated social networks of pregnant women (lay health consultant) **Medium**: Discussions with social network members	1. Pregnancy discussion networks about H1N1 vaccination 2. Discussants engaging in H1N1 vaccination conversations (Alters) and their 3. Educational status of the H1N1 vaccination discussants and supporters. 4. Social network size	1. Discussions about H1N1 vaccination influence vaccination uptake, and education plays a critical role in the flow of vaccination information among the participants. 2. Social network size: Participants with larger social networks were more likely to get vaccinated. 3. Homophily: Educated women preferred connecting with educated people with similar perceptions of H1N1 vaccinations.
13	Metcalf et al. 2013 [46]	Simulation modelling technique	Sample size -Not applicable, The number of participants ranged from 0-approximately 90 every month in the screening workshops.	Intragenerational: Experiential knowledge-	**Closed ties**: Friends **Loose ties**: Elderly people living close to each other participating in the *ElderSmile* dental health screening programme.	**Source**: Social ties- Friends **Medium**: Diffused social interaction-Word of mouth through object (mobile phone)	Two simulated models: 1. Agent-based model- Locating the proximity of the geographical locations of people participating in the ElderSmile dental health preventive screening programme to map the social network. 2. System dynamics model- A stock and flow model analysing the information diffusion patterns.	The simulation models demonstrated that dental healthcare-related information can be diffused across people through word of mouth.
14	Bradby et al. 2019- Quantitative component [40]	Questionnaire survey	*N* = 1755 UK = 318 Germany = 727 Portugal = 268 Sweden = 442	Intergenerational and intragenerational: Tacit and cultural knowledge	NA Reported in the qualitative component [Refer Table 5]	NA	The initial qualitative interviews of the residents reported five types of healthcare bricolage, further incorporated as variables during the survey to evaluate healthcare bricolage behaviour patterns in men and women. 1.No bricolage 2.Within system bricolage 3.Added-to-system bricolage 4.Alternative 5.Resources not used	Overall, women were more likely to engage in healthcare bricolage than men, highlighting the gender difference in healthcare-seeking behaviour.
15	Murakami et al. 2019 [34]	Questionnaire Survey	*N* = 4126	Not reported	Closed ties [Specific actors not reported]	**Source**: Particularly closed ties The mode of providing informational support has not been reported.	The study evaluated the association between: (1). Social relationship-social network and social support including informational support, and (2). Curative and preventive dental service utilisation	The analysis showed that informational support was not associated with the utilisation of curative and preventive dental care for both men and women.
16	Schafer 2013 [35]	A survey implementing interviews and post-interview questionnaires. The study evaluated egocentric social networks.	*N* = 3005	Intragenerational	**Close ties**: Partners, Children, Other kins (unspecified) **Loose ties**: Non-kin ties [non-kin not specified]	**Source**: Social network ties **Medium**: Discussion with network ties	(1). Visits to healthcare facilities to get examined by healthcare professionals (2). Conventional healthcare and CAM treatment utilisation (3). Social network: People (*Alters*) with whom participants discussed health-related matters, including service utilisation.	Discussing health-related matters with social network ties, particularly partners, was associated with increased physician visits and CAM utilisation.

**Intragenerational**: Between the same generation; **Intergenerational**: Across different generations

**Experiential knowledge**: From lived experiences; **Tacit knowledge**: Implicit in daily life practices

**Praxeological knowledge**: Information gained from inanimate objects (Bourdieu 1973); **Cultural knowledge**: Traditions and customs practiced for years giving rise to knowledge

**Table 5:** Qualitative studies

No.	Citation	Type of qualitative research method	Sample size	Type of healthcare knowledge searched or shared social networks [inherited, tacit, experiential, experientially similar, scientific/objectivist, praxeological]	Types of actors engaged in knowledge sharing [who is involved in information dissemination in the social circle: strong ties or loose ties - family, friends/peers, relatives, community]	Medium and source of knowledge sharing: [beliefs, culture, norms, daily life practices],	Key findings (The Influence of healthcare knowledge practices on people’s healthcare outcomes: Decision-making, healthcare visits, motivation to utilise services)
1	Gupta et al. 2015 [58]	In-depth interviews and focus group discussions were conducted with diverse participants	*N* = 253	**Intergenerational-**inherited tacit and experiential knowledge.	**Close ties**: Grandmother- daughters or daughters-in-law **Central actor**: Grandmothers were considered the gatekeepers and the chief source of information on various aspects of health and healthcare	**Source**: Strong ties **Medium**: Inheritance of lived experiences from mothers/mothers-in-law to Daughters/daughters-in-law	The knowledge of grandmothers helping their daughters or daughters-in-law who are young mothers of newborns in decision-making to visit healthcare centres when they or their children need treatment.
2	Comfort et al. 2022 [31]	Semi-structured in-depth interviews	*N* = 30 pregnant women	1.Intergenerational 2. Intragenerational: Experientially similar	**Close ties**: Mother Mother-in-law Grandmother Partner Sister Friends **Loose ties**: Women from the community, Neighbours Aunt Healthcare provider **Central actor**: Mothers and Mothers-in-law of pregnant women	**Source**: Strong and weak social network ties **Medium**: Cultural beliefs of mothers to provide advice and lived experiences of mothers of pregnancy influenced pregnant women to seek advice from them more than others in the social circle.	Women in this study reached out to strong ties-mothers and weak ties- neighbours and healthcare providers over strong ties like sisters or friends- for antenatal care advice.
3	Hill et al. 2018 [37]	In-depth interviews	*N* = 21	**1.Intergenerational**: Inherited tacit knowledge- 2. **Intragenerational-**Experiential knowledge	**Close ties**: 1. Family Members-Father-son 2. Friends in peer support groups	**Source**: Strong ties **Medium**: 1. Social interaction 2. Similar lived experiences of using HIV care	People living with HIV get informational support from their family members and peer support groups during HIV diagnosis, post-diagnosis treatment and mental health counselling services to cope with the impact of a positive HIV diagnosis.
4	Felsher et al. 2021 [29]	Qualitative approach to egocentric social network analysis with semi-structured interviews	*N* = 20	1.Intragenerational- Experientially similar 2.Intergenerational	**Close ties**: Friends, family members like step-sister, son, partners, **Loose ties**: Neighbours, sex clients **Central actor**: Friends	**Source**: Strong and loose ties **Medium**: 1. Interpersonal interactions in the social circle 2. Experientially similar- Women talking to other women at HIV risk about PrEP.	The intention of some women injecting drugs interacting with other women about PrEP to help them lower the HIV risk and increase their social ties to prevent social isolation;
5	Whitford et al. 2021 [50]	1. Focus group discussion 2. Semi-structured interviews with participants reluctant to participate in focus groups.	*N* = 61 [Focus group = 57 participants in 11 groups Interviews = 4]	Intragenerational	**Close ties**: Friends with similar experience [HIV + ve] **Loose ties**: Community outreach workers, volunteers, pimp, bar or nightclub managers	**Source**: Close and loose ties **Medium**: 1.Similar lived experiences of HIV testing of others. 2.Social skills and intention to help: Advocacy/bricolaging of pimps and managers by liaising with community centres for HIV testing and informing the sex workers	Most of the sex workers reported that they share or seek HIV-testing-related advice only from other HIV + ve workers, reluctant to interact with HIV -ve co-workers.
6	Threats 2020 Qualitative Component [45]	Semi-structured interview	*N* = 22	1. Intergenerational 2. Intragenerational: Experiential, scientific(objectivist) and praxeological knowledge	**Closed ties**: Family member-partners **Loose ties**: Healthcare professional	**Source**: Close and loose ties Media-Internet, television, newspapers **Medium**: Social interaction with people and passive learning from mass media information channels.	**HIV care-related information exchange**: Participants reported that they reach out to their network ties when they need HIV-care-related information for decision-making, and some participants reported that they usually do not explain everything in detail while sharing the information.
7	Ward et al. 2021 [44]	In-depth semi-structured interviews	*N* = 20	Intragenerational	**Closed ties**: Friends	**Source**: Closed ties **Medium**: Lived experiences of HCV testing Trust [catalyst for the interaction].	Participants interacted with members injecting drugs in their network to share HCV testing- and treatment-related information in different community settings.
8	Amoah et al. 2018 [30]	Semi-structured interviews	*N* = 79 residents [Urban = 28; rural = 51]	Intragenerational- Tacit knowledge	**Close ties**: Wife, brother, close friend **Loose ties**: Neighbours and acquaintances	Lived experiences of peers, Daily life communication with	People get recommendations about healthcare facilities, service types, and reasonable service costs from friends. Health access-related information is not sought exclusively but along with support and trust in urban and rural settings, unlike professional healthcare advice.
9	Brennan-Ing et al. 2023 [26]	Focus group and Interviews with people unable to participate in focus group due to Covid 19 restrictions	*N* = 77,	1.Intergenerational -experiential knowledge 2. Intragenerational-Tacit knowledge 3. Praxeological knowledge	**Close ties**: Daughter-Mother, Father-son, friend-to-friend, partners **Loose ties**: Community members	1. Social interactions in everyday life with family members, friends and community members at various events 2. Media-Information through television-praxeological 3. Lived experiences of vaccinations, some shared in the form of stories	Even after a surge in the misinformation about the risk of Covid-19 vaccinations circulated by the media and near ones in the social circle, older people decided whether to get vaccinated by talking to family, friends, and acquaintances.
10	Shahabuddin et al. 2019 [22]	1. In-depth interviews with adolescent girls, key informants 2. One focus group discussion with community health workers	*N* = 32	1. Intergenerational 2. Intragenerational	**Close ties**: Husbands, Mothers-in-law, **Loose ties**: Neighbours, community groups, health volunteers	Social interaction with different people and healthcare workers	The young girls receive information about maternal care from multiple sources, including mothers-in-law, neighbours, and community groups of women, which helps them mitigate their barriers and facilitates their use of antenatal and prenatal care.
11	Warren-Jeanpiere et al. 2010 [39]	Semi-structured interview using the interview guide	*N* = 17 daughters	Intergenerational	**Close ties**: Mother-daughter **Central actor**: Mother	**Source**: Mothers **Medium**: Social interaction is rooted in cultural beliefs about sexual and reproductive health.	Gynaecological healthcare communication between an Afro-American mother and daughter shapes how the daughter will communicate with the healthcare provider and her perceptions about healthcare services.
12	Kawaguchi et al. 2021 [23]	1. In-depth interviews of women and key informants 2. Focus group discussions	*N* = 16 [Healthcare facility delivery = 9 Home delivery = 7]	Intergenerational and Intragenerational	**Close ties**: Husband, parents, parents-in-law **Loose ties**: Relatives Health volunteers in villages **Central actor**: Women	**Source**: Social network ties **Medium**: Interpersonal communication across families and villages, Beliefs of women about the process of child delivery.	The study compared the interpersonal factors responsible for the decision-making of the place of child delivery- healthcare centre or home and found that families where women had more interaction with family members regarding the place of delivery, had a health centre delivery over home delivery.
13	Bradby et al. 2019-Qualitative component [40]	Ethnography: 1.Observation sessions 2. Semi-structured interviews	*N* = 160 residents	Intergenerational and intragenerational: Tacit knowledge cultural knowledge, and praxeological knowledge.	**Close ties**: Mother, Partner (wife) **Loose ties**: 1. Women acquaintances in extended community networks- Aunt 2. Health and social care professionals- GPs, nurses and social workers **Central actor-** Women in the community	**Source**: Social ties and mass media information channels **Medium**: 1. Communication with people in their social circle combined with individual’s cultural skills 2. Internet	**The technique of healthcare bricolage**: Healthcare bricolage refers to the phenomenon of people mobilising local resources available in their neighbourhoods to navigate a complex healthcare system.
14	Phillimore et al. 2019 [41]	Semi-structured interview	*N* = 2	Intragenerational	**Close ties**: Friends **Loose ties**: Yoga teacher, Non-governmental organisation (NGO)	**Source**: Social ties **Medium**: Providing advice through social communication	Two migrants to different European countries (Portugal and Sweden) were unable to access services in the new healthcare system; while the woman found informational support from a friend to consult a doctor from a private hospital, the asylum seeker was unable to access services even with financial support from the friend, and he eventually stopped interacting about health problems with others, became more secluded, resulting in **limited healthcare interaction**.
15	Samerski 2019 [42]	Ethnography: 1. Participant observation in the local A and E centre; 2. Narrative interviews using the topic guide as part of ethnography	*N* = 62, 42 interviews-Local residents 20- Healthcare professionals in the local community centres	**Intergenerational and intragenerational**: A combination of Experiential, tacit and praxeological knowledge	**Close ties**: Mother, friends, **Loose ties**: Acquaintances sharing common cultural and linguistic backgrounds. **Central actor**: NR	**Source**: Social network ties and mass media information sources **Medium**: 1. Lived experiences conveyed through social interaction, 2. Electronic/digital: internet. 3. Social skills of bricolaging multiple resources	People who face various challenges in using healthcare services acquire and co-produce healthcare knowledge through a bricolage of various resources in their social network, such as families, friends, and communities, who are also called **knowledge brokers** and mass media information sources, which illustrate people’s knowledge is multidimensional.
16	Foster et al. 2016 [43]	Ethnography- Netnography Covert, non-participant observation of the activities in an online community.	Not applicable [Analysis of online messages only]	Not reported	**Loose ties**: Online users	**Source**: Users **Medium**: Lived experiences built by formal education and social skills	People interacted online on a website to seek and share healthcare messages on various issues and get assistance from other users.
17	Celentano et al. 2021 [20]	Three focus groups, with each group comprising 9–11 mothers	Mother [*n* = 30] Adolescents [*n* = 136]	1. **Intergenerational**: Children to mother, and 2. **Intragenerational**: Friends-to-friends	**Close ties**: 1. Mothers, 2. Adolescent children and their school friends **Loose ties**: School **Central actor**: School-going adolescent children transmitting HPV-related information they learned in school from peers and staff to mothers	**Source**: Interplay of information between school and home 1. School acted as the source of HPV information for children where they interacted with peers 2. Mother’s beliefs on HPV drive their interaction with children	their adolescent children become more knowledgeable about HPV vaccination than their immigrant mothers by learning in school from friends, staff which they then share with parents after coming to home showing how HPV vaccination-related information diffused from school to home.
18	Woof et al. 2020 [59]	Semi-structured interviews with open-ended questions	[*N* = 19]	Intragenerational	**Close ties**: Family members such as sister-in-law, husband **Loose ties**: Community members	**Source**: Social network ties **Medium**: 1. Social interaction with women with lived experiences of going through the process of breast cancer screening 2. Cultural and religious beliefs of women about the health of the breasts. 3. Preconceived notions of women in healthcare service use	British-Pakistani immigrants reported experiencing cultural and linguistic barriers with different healthcare systems where interacting with someone from their community helps and encourages them to understand the process of breast screening and visit the GP.
19	McEwan et al. 2014 [24]	Semi-structured interview	*N* = 15	**Intragenerational**: Tacit and experiential knowledge.	**Close ties**: Family (Partner) Friends **Loose ties**: Neighbour **Central actor**: NR	**Source**: Social ties and Information Broadcasting sources-Television, newspaper, internet **Medium**: 1. Social interaction with family, friends and neighbours 2. Health beliefs around breast cancer 3. Information sources from related articles in newspapers, programs on television, and digital contents on the internet	Breast cancer service **knowledge co-production**: Women’s breast cancer diagnosis and treatment-related collective knowledge = Informal knowledge networks + information broadcasting sources + healthcare provider
20	Tatari et al. 2021 [47]	Combined qualitative methods: 1. Semi-structured focus groups- 5 2. Structured questionnaire group interviews- 2 3. Semi-structured individual interviews-	*N* = 37	**Intragenerational**: Scientific and praxeological knowledge.	**Close ties**: Friends **Loose ties**: 1. Acquaintances in the community with similar experiences of immigrating to a foreign country 2. The Neighbourhood Mother community centre.	**Source**: Friends **Medium**: A combination of lived experiences, of women attending cancer screening programmes and social networking sites like Facebook, YouTube, Instagram, and online WhatsApp groups	Immigrant women in Denmark recommended researchers arrange more screening programmes in local centres so that women can learn about the importance and purpose of cancer screening and pass information to other women who have limited access to information. This process is called the ‘**ripple effect**’.
21	Herrero-Arias and Diaz, 2021 [25]	1. Focus group discussions: Two focus groups with 6 and 4 participants 2. Interviews: A]. Individual interviews with 12 participants, B]. Couple interviews = 2 with 4 participants	*N* = 20	Intragenerational	**Strong ties**: Friends **Loose ties**: Relatives-Auntie Norwegian acquaintances A gynaecologist friend of a friend **Central actor**: Norwegian acquaintances	**Source**: Friends **Medium**: Social interaction	Southern-Eastern immigrants from Europe immigrated to Norway and, facing various challenges in comprehending the new Norwegian healthcare system, reached out to other immigrant friends and Norwegian acquaintances to receive appropriate information about health services.
22	Izquierdo et al. 2018 [49]	Engagement workshop with open-ended questions	*N* = 15 12 veterans, 3 family members participating in more than 1 workshop.	Intragenerational	**Close ties**: Friends **Loose ties**: Fellow veterans, Healthcare provider (Psychiatrist) **Central actor**: Not reported	**Source**: Friends **Medium**: Lived healthcare experiences of veterans	Veterans mentioned they wanted to share their lived experiences of accessing VA (Veteran Affairs) healthcare services with other veterans to help them access VA healthcare.
23	Madden 2015 [28]	1-year ethnographic study: 1.10 h observation at USA-Mexico border immigration areas 2.>100 h in-depth interviews	*N* = 59 (1)0.44 patients and caregivers travelling across USA-Mexico borders for prescription drugs, including undocumented immigrants to the USA (2)0.15 healthcare professionals from community centres	**Intragenerational and** **Intergenerational**: Tacit knowledge	**Close ties**: Family-Granddaughter-Grandmother Friends Partner	**Source**: Strong ties **Medium**: Cultural health capital, Familial capital -knowledge and skills, navigational capital, and Linguistic capital	The author observed people mobilised various assets from their community’s cultural wealth to provide optimal healthcare to their loved ones.
24	Townsend et al. 2014 [61]	In-depth individual interviews	*N* = 37	**Intra-generational and inter-generational** Experiential knowledge and Praxeological knowledge-	**Close ties**: 1. Family- Mothers, fathers, daughters, sisters, partners 2. Friends **Loose ties**: 1. Co-workers 2. Community members 3. Family physicians or other healthcare professionals	**Source and Medium**: 1. Social interaction with people in social circles, combined with 2. Intergenerational beliefs about seeking healthcare consultations- Women’s beliefs about the severity of illness and preference for self-management over professional care and 3. Rheumatoid Arthritis-related information sources available on the internet as a tool to cross-verify medical advice.	Women sought medical advice after people in their social circle motivated them to seek medical advice and get a quicker referral to a rheumatologist, along with instrumental support from family, friends, co-workers, and healthcare professionals.
25	Kettle et al. 2019 [36]	Interviews	*N* = 43	**Intergenerational**: Experiential knowledge	**Close ties**: Parents- mostly mother, father, Grandmother **Loose ties**: School dentist	**Source and medium**: Close ties: Parents and grandparents	1. Mothers play an essential role in developing children’s habit of regular dental visits. 2. Dentists visited the school to check students’ oral health, which helped students learn about the importance of dental checkups and the need to visit the dentist. 3. Grandparents wanted to pass their knowledge to grandkids about different aspects of oral health, including dental visits.

**Intra-generational**: Between the same generation; **Inter-generational**: Across different generations; **Experiential knowledge**: From lived experiences; **Tacit knowledge**: Implicit in daily life practices; **Praxeological knowledge**: Information gained by inanimate objects (Bourdieu 1973); **Cultural knowledge**: Traditions and customs practiced for years giving rise to knowledge

*NR* Not reported

People’s social network ties (close and loose) served as the actors/mediators engaged in knowledge-seeking or sharing. They were found to be the most prevalent source of healthcare knowledge across both urban and rural communities. In 20 studies (4 quantitative and 16 qualitative), participants acquired health service-related knowledge from both close and loose ties, whereas in 11 studies (5 quantitative, 7 qualitative) loose ties were the principal source of health service-related knowledge. Friends were found to be the most predominant source of knowledge in 23 studies, followed by various family members in 15 studies (Tables 4 and 5). Loose ties comprised a wide variety of community members, more distant relatives, coworkers, community and healthcare professionals and institutions like community centres, schools and hospitals, of which healthcare professionals emerged as the most predominant contact in three studies. Only 9 studies (2 quantitative, 7 qualitative) reported about the chief actor or mediator of healthcare knowledge in communities, out of which, in 6 studies, women of different age groups and generations emerged as the most prevalent central actor.

The studies that were included did not explicitly characterise the forms of healthcare knowledge that emerged in local and online communities. Nevertheless, it can be inferred from the findings that lay people searched, acquired, co-constructed and shared diverse forms of knowledge, predominantly during informal social interactions in neighbourhoods, support groups, various organisations and with healthcare professionals within the generations (*intra-generational*) and across different generations (*inter-generational*), which can be further grouped as familial, peer-to-peer, and parental, with maternal being more prevalent across the studies. These interpersonal healthcare interactions with formal and informal social network ties can be face-to-face and online. People actively and passively learned about different healthcare services predominantly from the lived experiences of others (*experiential knowledge*) in their social circles, as shown in fourteen studies, including five studies where the same healthcare experiences led people to connect and exchange healthcare information (*experientially similar knowledge*).

Healthcare interactions were found to be driven by people’s cultural practices, beliefs, social norms, customs, and pre-conceived notions about health passed down through generations, which may give rise to *tacit* and *cultural knowledge* implicit in people’s everyday lives, as noted in seven studies. Sharing healthcare knowledge gained by formal education or attending educational or promotional programmes (*scientific/objectivist knowledge*) was noted in one study. Moreover, one study [46] reported that *word of mouth* was a medium through which dental screening-related knowledge was diffused across a specific urban region. Along with healthcare interactions, people acquired knowledge from mass media sources broadcasting healthcare service-related information (*praxeological knowledge*) to a large population in eight studies where the internet, television and newspapers were the commonly used sources, with the internet gradually becoming an easily accessible and essential source [45]. The type of healthcare knowledge could not be inferred in three studies [27, 34, 43].

Findings showed how some factors encouraged or discouraged people to seek or share healthcare knowledge. Studies revealed that not everyone in local and online communities sought or shared healthcare knowledge. Eleven studies showed that people’s trust acted as a catalyst when seeking healthcare knowledge and tended to reach out to trusted network ties because privacy and confidentiality were crucial for them. The most prominent phenomenon identified was homophily, where people wanted to connect with people with similar experiences and backgrounds to obtain or receive vaccination-related information. Some studies reported that people reached out to others in their social circle because, along with healthcare knowledge, they also received essential emotional and instrumental support such as financial help and transportation. Two studies showed that some people, especially the elderly, believed in helping the people of the community as their social responsibility, motivating them to share healthcare knowledge. However, for several reasons, some people were found unwilling to interact with others about different aspects of healthcare in their social circles. In contrast to the informal knowledge networks, ten studies highlighted people’s resistance to seeking or sharing information across their networks due to pre-conceived notions, taboos and health beliefs, cultural norms, social practices, and willingness to share information.

## Discussion

It is clear from the review findings, that the phenomenon studied is a complex one and relatively ill defined. This paper therefore provides some structure as to understanding the state of evidence in this area and where the gaps in research may fall. Although, the review identified a wide range of sociological theories and concepts, it is important to note that several studies used similar theories such as the social ecological model (which was applied in 3 studies). The theoretical underpinning of the phenomenon studied also often involved overlapping theories. For instance, *diffusion of information* in Celentano et al. 2021 [20] and the *ripple effect* in Tatari et al. 2021 [47] - which both help to understand the same social phenomenon of passing information to others in the social network. These theories can be used to understand how health-service-related knowledge might spread across social networks in local and online communities at micro, meso and macro levels. However, a distinction between health and healthcare knowledge was found not to be distinguishable in most theories. Rather, ‘health knowledge’ appears to be used as an umbrella term encompassing knowledge concerning well-being, illness, self-treatment, and healthcare services. A theoretical framework exclusively focussing on lay people’s healthcare service-related knowledge seeking and sharing was not found.

The tradition of healthcare knowledge seeking and sharing was found to be prevalent in both developed and developing countries in urban, suburban, rural or online communities among populations of different age-groups, genders, ethnicities and generations in varying social, cultural, and economic circumstances for different healthcare services, which included life-threatening conditions like cancer and emotionally challenging mental illness which increased during adverse healthcare circumstances. This review found that although healthcare professionals were an essential source of information in healthcare settings, people’s journey from the home or community to the healthcare facility was shaped by the knowledge they gained from informal sources. Findings align with a previous review by Konstantinou et al. 2021 [32] which found that people’s attitudes, behaviour and decision-making of using immunisation services were shaped by their discussions with informal and formal social network ties, including healthcare providers. Our findings concur with previous studies [4, 6, 7] that show that people’s health knowledge is not an individual possession but distributed in social networks; and that *‘Bourdieu’s three types of theoretical knowledge*’, which describes how people derive knowledge from surrounding people and objects [62] are pertinent for people’s health service-related knowledge. Furthermore, the interpersonal healthcare discussions in the studies imply that groups of people engage in shared decision-making to use services, which resonates with the concept of Rapley’s *‘distributed decision making’* [63], elucidating that people gain knowledge about health from multiple informal sources which shape their decision making, unlike the conventional clinical/medical decision making-a dyadic communication between doctors and patients driven by cognitive knowledge of disease and treatment.

Although the included studies showed some evidence of healthcare knowledge flow among people at inter-personal and community levels, relatively few studies (*n* = 11/41) have investigated the phenomenon of people attempting to gather, provide and discuss healthcare information as *‘knowledge*’. At interpersonal and community levels, people seeking or sharing healthcare information across their social circles has often been described as mere ‘communication’, ‘discussion’ or ‘support’ without recognising that the healthcare information contained within people’s lay experiences and narratives when transmitted through discussions make others more knowledgeable. While professionals and stakeholders communicating healthcare information within institutional settings among scientific communities is called knowledge exchange and transfer [64], healthcare information flow in different communities of lay people is not elucidated with an equal amount of conceptual clarity. This interpretation resonates with the work of Muscat et al. 2022 [8] who reviewed the literature on distributed health literacy and found that shared health knowledge is conceptually and empirically under-developed and has not been differentiated from other types of social support.

Hence, it can be inferred from the included studies that the concept of people’s healthcare knowledge at the interpersonal level is not well defined. This review, therefore, suggests a need to distinguish people’s healthcare knowledge gained from local resources in everyday lives from expert knowledge, as suggested by Popay 1998 [11], where knowledge relating to healthcare services should be viewed separately from knowledge of general health, self-treatment and well-being as it is essential to understand the impact of healthcare knowledge flow on people’s behaviour of utilising services and their health outcomes. Our findings show that various forms of lay healthcare knowledge are acquired or provided by people across social networks across generations at the meso-level through different mechanisms which collaboratively form lay people’s *healthcare knowledge practices*. Therefore, people’s *healthcare knowledge practices* can be understood as a combination of:

1. **Healthcare knowledge co-construction/co-production/co-creation** occurs when individuals accumulate health service-related knowledge from social network ties in combination with information broadcasting objects like television, newspapers, and the internet [24, 42, 50].
2. **Healthcare knowledge sharing/exchange/transfer** occurs when an individual from a social circle provides health service-related knowledge to another who gains knowledge [37–39, 44, 47].
3. **Healthcare knowledge diffusion/dissemination/transmission** occurs when health service-related knowledge is shared with more than one person or group, who then further spreads it to a larger number of people, for example, through word-of-mouth [20, 38, 46].
4. **Healthcare knowledge suppression** occurs when people in a community are reluctant to seek or share health service-related knowledge with others due to various reasons, including fear of judgement, stigmatisation, cultural beliefs, privacy, and embarrassment [29, 45, 50, 59, 60].

Lay people’s multi-dimensional knowledge practices in diverse communities identified in this study present a more expansive view of healthcare knowledge at the interpersonal level in comparison to previous work [65–68]; which primarily evaluates an individual’s scientific knowledge about the disease, treatment, medical adherence and frequency of accessing healthcare services without taking into account the knowledge people gain from informal sources around them through their agency and their thoughts and feelings about using healthcare services as highlighted in this review.

### Mapping evidence gaps

Most included studies did not focus exclusively on the distributed nature of people’s healthcare knowledge at the meso-level but it was more of a secondary or minor focus. This leaves scope for more specific study of this phenomenon. Moreover, since study methods were predominantly standardised questionnaires and semi-structured interviews, with only one clinical trial design and no studies using innovative qualitative methods such as visual and creative methods, there is a need to build up the evidence using methods providing a richer and more nuanced study. Whereas a few quantitative studies employed Social Network Analysis (SNA), all of these used the ego-centric approach, and none used the socio-centric approach which focuses on people’s interactions and relationships within a group or community, which is a further gap. Moreover, while 4 studies used quantitative SNA, only 1 study of these used a qualitative approach. Further gaps in the literature concerned the study of socially isolated or marginalised communities. Although there were a few studies on immigrants of diverse ethnicities, only one study involved unauthorised immigrants. There were no studies exploring the healthcare knowledge practices of homeless people, asylum seekers, specially-abled and socially isolated people and only 2 studies involving transgender people and people with different sexual orientations. Although socio-economic status and education level is reported in many studies, with a range of income status and education level included among participants, the specific role of education in shaping the extent of intra and inter-generational knowledge seeking and sharing is unexplored because the primary studies lack the specific focus on the phenomenon needed. A further gap is the relative dearth of studies which are focused on healthcare knowledge sharing among children– there were no studies undertaken with participants aged under 14-years. Whereas studies of people’s knowledge practices around a diverse range of healthcare services have been undertaken, there are relatively few or no studies on chronic degenerative, life-threatening diseases which significantly impact the quality of life such as dementia. For all types of healthcare services, authors found evidence of knowledge seeking or sharing among people, although one study reported conflicting findings and showed that informational support was *not* associated with participants’ dental service utilisation, and this which needs further research. Many studies reported the central role of women as knowledge mediators, specifically mothers, but very limited data was found on the role of men so more studies would be helpful to understand the gender variation in healthcare knowledge practices.

### Strengths and limitations

Every electronic search is a balance between sensitivity (able to pick up all relevant studies) and specificity (precision) [69]. Because the phenomenon under study was relatively conceptually indistinct, the search was necessarily sensitivity and led to over 9,000 abstracts to be screened. Nevertheless, because of the narrow nature of inclusion criteria, which focused on the meso-level, only 41 articles were included, which is relatively small compared to some scoping reviews. This indicates that the area is under-studied, although since the search was restricted to English language due to time and resource (cost of language translations) constraints, this may have impacted inclusiveness relating to some settings. On the other hand, the literature identified included studies from 15 different countries, including 9 from lower-middle and lower-income countries, and so the limitation may have not compromised inclusivity significantly. Gaps in the literature have been highlighted above, and a consequence of some of the gaps in the literature e.g. that few studies focus exclusively on healthcare knowledge sharing at the meso-level, and there are no studies on children under 14 years of age, is that presenting findings according to sub-groups such as age, is not possible. This is a limitation, because children/adolescents may have very different experiences than adults, especially given their different social networks and use of social media. Likewise, the influence of educational level in how knowledge is shaped and shared at the meso-level is very relevant, but there is insufficient data to be able to draw out any conclusions.

## Conclusion

People in social networks are exposed to diverse lived experiences and stories of healthcare encounters when they; search, interact, discuss or provide advice to family, friends, relatives, colleagues, community members across generations, receive informational support from local organisations and acquire knowledge from information broadcasting sources. These efforts enable people to co-construct, share, and disseminate multiple forms of healthcare knowledge, which collectively shapes the shared healthcare knowledge of the community. Therefore, people’s healthcare knowledge developed outside health institutions in local and virtual environments can be *multi-dimensional* which can be viewed as an amalgamation of lived experiences, beliefs, culture, heritage, social norms and practices reinforced with trust, emotional and instrumental support which diverges from the scientific knowledge of experts rooted heavily in objectivity. The review shows that healthcare professionals are not the sole sources of healthcare information for lay people. For this reason, this review proposes that healthcare professionals developing health literacy interventions should consider individuals as constituents of the social network system rather than solely as solitary elements disconnected from the social world. Social networks and support factors should be included when considering how to design and implement public health programmes to improve people’s knowledge about healthcare services.

## Supplementary Information

Supplementary Material 1. Supplementary Tables 1, 2 and 3 and amendments to the search strategy.

## Data Availability

All data generated or analysed during this review are included in this article [and its additional information files] and are available from the corresponding author upon request. The search strategy has been deposited to searchRxiv, searchRxiv - Submit a search| CABI Digital Library and available at: CINAHL: 10.1079/searchRxiv.2024.00762. Ovid MEDLINE-R: 10.1079/searchRxiv.2024.00763. Web of Science: 10.1079/searchRxiv.2024.00764. APA PsycInfo: 10.1079/searchRxiv.2024.00766.

## Abbreviations

CRC: Colo-Rectal Cancer
Covid-19: Coronavirus Disease 2019
IMD: Index of Multiple Deprivation
MeSH: Medical Subject Headings
NR: Not reported
NLS: New Literacy Studies
HIV: Human Immuno-deficiency Virus
HPV: Human Papilloma Virus
H1N1: Influenza A virus subtype
RCT: Randomised Controlled Trial
