# Supplementary material for: Knowledge-seeking and knowledge sharing of health services across social networks and communities: a scoping review
Source: BMC Health Serv Res. 2025 Mar 27;25:443. doi: 10.1186/s12913-025-12525-y (PMC11948718; doi:10.1186/s12913-025-12525-y)
Supplement: Supplementary file 1 — Supplementary Material 1. Supplementary Tables 1, 2 and 3 and amendments to the search strategy. [file 12913_2025_12525_MOESM1_ESM.docx]

**Appendix:**

**Supplementary tables:**

**Supplementary Table 1:** **Scoping review framework adapted from the enhanced scoping review framework of Levac et al 2010.**

| Arksey and O’Malley’s traditional framework [2005] | Shortcomings of Arksey and O’Malley’s framework addressed by Levac et al 2010 | Levac et al 2010 advanced framework with enhancements in Arksey and O’Malley scoping review framework | Adaptation of **Levac et al 2010** framework for this scoping review with additional adjustments relevant to the review question and the primary research topic: |
| --- | --- | --- | --- |
| **Step 1: Research question development** | 1.The purpose of scoping review is not considered while formulating the question  2. The process of formulating the research question is not clearly described in Arksey and O’Malley framework. | **Developing the research question and purpose of the scoping review:** The important criteria is to formulate a broad research question | This preliminary step was to formulate the research question:  1. Initial informal search about the topic.  2. To set out the rationale of the scoping review  3. Analysis of the articles obtained in the preliminary search relevant to the research domain.  4. Formulate the research question by integrating the target population, phenomenon of interest and study outcomes using different frameworks.  5. To determine the primary aim and objectives of the scoping review. |
| **Step 2: Identifying relevant studies** | Lack of understanding on how to balance extent and comprehensiveness while conducting the review when evidence base is large and complex with varied information. | Levac et al divided this stage into 3 steps:  1.Define the scope of the review according to the review question and purpose.  2.Recruitment of reviewers to conduct the scoping review.  3.Report potential limitations of the review and explain the underlying reasons, when defining scope of the review is difficult. | The scoping review frameworks devised by Arksey and O’Malley 2005 and Levac et al 2010 have not explained the process of developing the search strategy for scoping reviews. The decision-making process of selecting the key search terms and databases and developing and running a search strategy across different databases are not clearly explained in these two frameworks.  This step included developing a search strategy to locate the relevant literature:  1. Determining key search terms from the initial articles considering the research question.  2. Developing a search string algorithm by combining the search terms appropriately.  3. Selecting relevant databases to run the search.  4. Running the search terms across different electronic databases to map the MeSH terms (for Medline) and subject headings (for CINAHL and PsycINFO) and include them in the search strategy.  5. Documenting all the changes made in the search strings in different databases, which included the application of different (a) Boolean and proximity operators, (b) truncation and wild cards, (c)commands and re-running the search. |
| **Step 3.**  **Selecting relevant studies** | Arksey and O’Malley framework has not described this stage comprehensively. The process of determining a study's relevance in relation to the review question needs further precision. | Levac et al 2010 divided this stage into five steps:  1. The process of study selection should be iterative. The search strategy should be refined iteratively based on the initial screening of the articles.  2. Determining the inclusion and exclusion criteria.  3. Reviewers should discuss the abstract screening process at the beginning, mid-point and concluding phases to mitigate any challenges faced during the study selection.  4. Two reviewers should independently review the included studies.  5. Including a third reviewer to resolve any disagreement between the two reviewers. | The process of determining inclusion and exclusion criteria and the approach to title-abstract screening are not explained clearly in the amendments proposed by Levac et al 2010:  This step included:  1. Eligibility criteria were developed**:** Determining inclusion and exclusion criteria using the SPIDER framework.  2. Title and abstract screening with the help of pre-determined eligibility criteria followed by full-text screening of selected studies.  3. Double screening: To maintain rigour, the second reviewer independently reviewed a proportion of the abstracts, with both reviewers blinded.  4. Full-text screening was conducted using the eligibility criteria to shortlist studies for the review. |
| **Step 4**: **Data charting** | The extent and process of data extraction from included studies is poorly defined in the Arksey and O’Malley framework. | Levac et al 2010 divided this stage into several iterative steps**.**  1.Developing a data extraction chart to determine relevant variables to extract from the included studies.  2. Data extraction in an iterative process.  3. Consider the qualitative content analysis of the data from included studies.  3. Two independent reviewers should conduct data extraction for the first few included studies using the data extraction table, followed by a discussion about the consistency of the data extraction process in relation to the research question and objectives. | This step included:  1. Data extraction tables were developed to synthesise the key concepts informing the research topic, aims and objectives in the scoping review protocol.  2. The data extraction tables were developed iteratively.  3. To maintain rigour, a second reviewer conducted data extraction independently using the data extraction tables for the first few studies and discussed the relevance of the extracted data to the review topic. |
| **Step 5: Data reporting** | The traditional framework did not describe the process in detail. Levac et al 2010 divided this stage into 3 distinct steps. | Data analysis of the included studies and reporting of the results:  1. Analysis (including qualitative thematic analysis)  2. Reporting the results.  3. Interpretation of the study findings concerning the review topic and purpose; reporting the research gap and implications for practice and policy | This step included data integration:  1. Collating and summarising the data.  2. Narrative description of the key concepts identified in the included studies.  3. Mapping and identifying research gaps for further research. |

**Supplementary Table 2:**

The research question has been formulated considering the target population, phenomenon of interest and study outcome in mind:

(1). The target population; (2).The phenomenon of interest, and (3).The study outcome of the review has been integrated and applied to two frameworks, [1] **SPIDER** and [2] **PICo-qualitative,** to construct the research question as shown below:

**Supplementary table 2: Illustration of formulating the research question using SPIDER and PICo frameworks.**

| **SPIDER Framework** | |  | **PICo Framework** | |
| --- | --- | --- | --- | --- |
| **S-sample** | Local communities comprised of family, friends and community members connected by social ties |  | **P-Population** | Local communities comprised of family, friends and community members connected by social ties |
| **PI-Phenomenon of interest** | Knowledge and belief acquisition, co-construction or exchange amongst family members, friends and community members through various mediums |  | **I-**Phenomenon of **Interest** | Knowledge and belief acquisition, co-construction or exchange amongst family members, friends and community members through various mediums |
| **D-Design** | Literature review |  | **Co-Context** | Influence on healthcare service utilisation |
| **IE-Evaluation** | Influence on healthcare service utilisation |  |  |  |
| **R-Study type** | Empirical, theoretical and review studies including grey literature |  |  |  |
|  |  |  |  |  |
|  |  |  |  |  |

**How are knowledge and beliefs acquired, co-constructed, exchanged and adapted in local communities influencing decision making of people to help themselves, their families and community members to access healthcare services?**

**Supplementary Table 3: The search string algorithm developed to retrieve relevant studies from databases**

| Target population AND | Phenomenon of interest AND | Study outcome |
| --- | --- | --- |
| ‘Local community’ OR Neighbourhood OR Neighborhood OR Localities OR Locality OR Locale OR Family OR Familsm OR Familismo OR Friends OR Peers OR Relatives OR People OR group OR Generation OR Inter-generational OR Inter-personal OR Parents OR Mother OR Father OR Meso-level OR ‘Social network’ OR ‘Social ties’ OR ‘network ties’ OR ‘Social relations’ OR ‘Social relationships’ OR ‘Social contacts’ OR ‘Patient’ | ‘Knowledge construction’ OR ‘Knowledge co-construction’ OR ‘Knowledge co-production’ OR ‘Knowledge Co-produce*’ OR ‘Knowledge creation’ OR ‘Knowledge co-creation’ OR ‘Experiential knowledge’ OR ‘Experiential* OR ‘experience’ OR ‘Knowledge sharing’ OR ‘Knowledge exchange’ OR ‘Knowledge exchange*’OR ‘Shared knowledge’ OR ‘Knowledge practices’ OR ‘Narrative knowledge’ OR ‘Perceived knowledge’ OR ‘Lay knowledge’ OR ‘Local knowledge’ OR ‘Indigenous Knowledge’ OR ‘Tacit Knowledge’ OR ‘Lived experience’ OR ‘Shared experience’ OR ‘Storytelling’ OR ‘Story building’ OR ‘Storytelling interventions’ OR ‘Story sharing’ OR Narrative OR Narration OR ‘Narrative interventions’ OR ‘Health beliefs’ OR ‘Values’ OR Norms OR ‘Discussion networks’ OR ‘interactions’ OR Conversations OR ‘Community health literacy’ OR ‘Distributed health literacy’ OR ‘health literacy as social practice’ OR ‘Information sharing’ OR ‘Information exchange’ OR ‘Information flow’ OR ‘health* related information’ OR ‘Information flow’ OR ‘ information diffusion’ OR ‘Bricolage’  **NOT** (Doctor* or Dentist* or Nurse* or Clinician* or Professional* or Practitioner* or Healthcare professional*) | ‘Health care’ OR ‘Healthcare’ OR ‘Medical care’ OR ‘Healthcare service’ OR ‘health service’ OR ‘General practice’ OR hospital OR ‘Dental practice’ OR ‘Pharmacy’ OR ‘Screening’ OR ‘Physician’ OR Doctor OR Dentist  **AND**  ‘Access’ OR ‘Utilisation’ OR ‘Utilisation’ OR Uptake OR Visits OR visiting OR Attendance OR attending OR Use OR ‘behavior’ OR ‘Behaviour’ OR ‘Decision-making’ |

**The search strategy was amended across different databases applying appropriate Mesh terms (for Medline), subject headings (for CINAHL and APA PsycINFO), proximity and boolean operators:**

**[1]. MEDLINE**

Ovid MEDLINE(R) ALL <1946 to 2024>

1 local communit*.mp. [mp=title, book title, abstract, original title, name of substance word, subject heading word, floating sub-heading word, keyword heading word, organism supplementary concept word, protocol supplementary concept word, rare disease supplementary concept word, unique identifier, synonyms, population supplementary concept word, anatomy supplementary concept word] 10310

2 Neighbourhood*.mp. 10217

3 Neighborhood*.mp. or exp Neighborhood Characteristics/ 36382

4 Famil*.mp. or exp Family/ 1788832

5 Familism.mp. 497

6 Familismo.mp. 133

7 exp Family Relations/ or Family relation*.mp. 125581

8 exp Interpersonal Relations/ or exp Friends/ or Friend*.mp. 502648

9 exp Peer Group/ or peer*.mp. 163826

10 interpersonal relation*.mp. 85177

11 social ties.mp. 1370

12 social network*.mp. 30823

13 community network*.mp. or exp Community Networks/ 8271

14 Network ties.mp. or exp Social Networking/ 6693

15 Patient.ti,ab,kw. 2938585

16 (intergeneration* adj3 relation*).mp. [mp=title, book title, abstract, original title, name of substance word, subject heading word, floating sub-heading word, keyword heading word, organism supplementary concept word, protocol supplementary concept word, rare disease supplementary concept word, unique identifier, synonyms, population supplementary concept word, anatomy supplementary concept word] 5185

17 (Generation* adj3 relation*).mp. [mp=title, book title, abstract, original title, name of substance word, subject heading word, floating sub-heading word, keyword heading word, organism supplementary concept word, protocol supplementary concept word, rare disease supplementary concept word, unique identifier, synonyms, population supplementary concept word, anatomy supplementary concept word] 1013

18 1 or 2 or 3 or 4 or 5 or 6 or 7 or 8 or 9 or 10 or 11 or 12 or 13 or 14 or 15 or 16 or 17 5067364

19 (Knowledge adj1 construct*).ti,ab,kw. 532

20 (Knowledge adj1 co-construct*).ti,ab,kw. 34

21 (Knowledge adj1 co-produc*).ti,ab,kw. 121

22 (Knowledge adj1 co-creation).ti,ab,kw. 24

23 (Knowledge adj1 shar*).ti,ab,kw. 3416

24 (Knowledge adj3 exchange).ti,ab,kw. 2016

25 (Knowledge adj1 experiential).ti,ab,kw. 766

26 ((narrative or lay or Local or indigenous or Personal or Traditional or Inherit* or Familial or Perceived) adj1 knowledge).ti,ab,kw. 6905

27 ((Lived or Shared) adj1 experience*).ti,ab,kw. 16409

28 (Knowledge adj1 disseminat*).ti,ab,kw. 1166

29 (Storytelling or "Story telling" or "Storytelling intervention" or "Story build*").ti,ab,kw. 3064

30 (shar* adj2 stor*).ti,ab,kw. 1830

31 narrative*.ti,ab,kw. 96150

32 Discussion network*.ti,ab,kw. 72

33 "Community health literacy".mp. [mp=title, book title, abstract, original title, name of substance word, subject heading word, floating sub-heading word, keyword heading word, organism supplementary concept word, protocol supplementary concept word, rare disease supplementary concept word, unique identifier, synonyms, population supplementary concept word, anatomy supplementary concept word] 30

34 "Distributed health literacy".ti,ab,kw. 17

35 health literacy as a social practice.ti,ab,kw. 1

36 (information adj4 exchange).ti,ab,kw. 9284

37 (diffusion adj3 information).ti,ab,kw. 1097

38 bricolage.ti,ab,kw. 135

39 Knowledge practice*.ti,ab,kw. 1323

40 ((Communit* or Famil* or Friend* or intergeneration* or generation*) adj4 Knowledge).ti,ab,kw. 14033

41 (19 or 20 or 21 or 22 or 23 or 24 or 25 or 26 or 27 or 28 or 29 or 30 or 31 or 32 or 33 or 34 or 35 or 36 or 37 or 38 or 39 or 40)

NOT (Doctor* or Dentist* or Nurse* or Clinician* or Professional* or Practitioner* or Healthcare professional*).mp. [mp=title, book title, abstract, original title, name of substance word, subject heading word, floating sub-heading word, keyword heading word, organism supplementary concept word, protocol supplementary concept word, rare disease supplementary concept word, unique identifier, synonyms, population supplementary concept word, anatomy supplementary concept word] 114931

42 ((access* or utilisation or utilisation or uptake) adj3 ("Health* service*" or "Health care service*" or healthcare or "health care" or "general practice*" or Screening)).ti,ab,kw. 81624

43 ((access* or Utilisation or Utilization or Uptake) adj3 ("Primary care" or "Secondary care" or "Tertiary care" or "Dental care" or "Dental service*" or "Dental health* service*" or "Oral health care" or "Oral health* service*" or "Advanced care" or Hospital* or Doctor* or Physician* or Dentist*)).ti,ab,kw. 22460

44 (navigat* adj3 ("health care" or healthcare)).mp. [mp=title, book title, abstract, original title, name of substance word, subject heading word, floating sub-heading word, keyword heading word, organism supplementary concept word, protocol supplementary concept word, rare disease supplementary concept word, unique identifier, synonyms, population supplementary concept word, anatomy supplementary concept word] 1357

45 health-care decision-making.ti,ab,kw. 910

46 Patient acceptance of health care.mp. or exp *"Patient Acceptance of Health Care"/ 117830

47 (facilities and services utilisation).mp. [mp=title, book title, abstract, original title, name of substance word, subject heading word, floating sub-heading word, keyword heading word, organism supplementary concept word, protocol supplementary concept word, rare disease supplementary concept word, unique identifier, synonyms, population supplementary concept word, anatomy supplementary concept word] 1555

48 health services accessibility.mp. or exp Health Services Accessibility/ 142096

49 (Appointments and schedules).mp. [mp=title, book title, abstract, original title, name of substance word, subject heading word, floating sub-heading word, keyword heading word, organism supplementary concept word, protocol supplementary concept word, rare disease supplementary concept word, unique identifier, synonyms, population supplementary concept word, anatomy supplementary concept word] 10297

50 ((Health* or "Health care" or service) adj2 Delivery).ti,ab,kw. 48552

51 (influenc* or shap* or impact*).mp. [mp=title, book title, abstract, original title, name of substance word, subject heading word, floating sub-heading word, keyword heading word, organism supplementary concept word, protocol supplementary concept word, rare disease supplementary concept word, unique identifier, synonyms, population supplementary concept word, anatomy supplementary concept word] 4039593

52 (Health* behavi*r or "Health care behavi*r").mp. [mp=title, book title, abstract, original title, name of substance word, subject heading word, floating sub-heading word, keyword heading word, organism supplementary concept word, protocol supplementary concept word, rare disease supplementary concept word, unique identifier, synonyms, population supplementary concept word, anatomy supplementary concept word] 71282

53 51 and 52 19220

54 ((Health* or "Health care" or Physician*) adj3 (visit* or behaviour)).mp. [mp=title, book title, abstract, original title, name of substance word, subject heading word, floating sub-heading word, keyword heading word, organism supplementary concept word, protocol supplementary concept word, rare disease supplementary concept word, unique identifier, synonyms, population supplementary concept word, anatomy supplementary concept word]

55 42 or 43 or 44 or 45 or 46 or 47 or 48 or 49 or 50 or 53 or 54

56 18 and 41

57 55 and 56

58 limit 57 to English language

59 limit 58 to yr="2003 - 2024"

**[2]. WEB OF SCIENCE:**

“local communit*” OR “neighborhood*” OR “neighbourhood*” OR “Famil*” OR Familism OR Familismo OR “Friend*” OR Peer* OR “Community support” OR “Generation*” OR “Interpersonal relation*” OR “Intergenerational relation*” OR “Social ties” OR “Social group” OR “Social network*” OR “community network*” OR “Network ties” OR “Community group” OR “family relation*” OR “Parent*” OR “Mother*” OR “Mother child relation*” OR “Intergeneration* NEAR/3 relation*” OR “Generation NEAR/3 relation*”

**AND**

“knowledge construct*” OR “knowledge co-construct*” OR “knowledge co-produc*” OR “knowledge creation” OR “knowledge co-creation” OR “knowledge exchange” OR “Knowledge shar*” OR “Experiential knowledge” OR “narrative knowledge” OR “lay knowledge” OR “local knowledge” OR “Personal knowledge” OR “Traditional knowledge” OR “Inherit* Knowledge” OR “Familial knowledge” OR “indigenous knowledge” OR “knowledge dissemination” OR “perceived knowledge” OR “Lived experience*” OR "Shared experience*" OR "healthcare experience*" OR "health care experience*" OR “Storytelling” OR "story telling" OR "Story building" OR “storytelling intervention” OR “Narration” OR “narrative intervention” OR “health* belief*” OR “Health care belief*” OR “discussion network*” OR "community health literacy" OR “distributed health literacy” OR “health literacy as a social practice” OR “Healthcare Information Exchange” OR “healthcare advice” OR “Health* bricolage” OR “Personal narrative*” OR "knowledge practice*" OR “Communit* NEAR/4 Knowledge” OR “famil* NEAR/4 knowledge” OR “friends NEAR/4 knowledge” OR “intergenerational NEAR/4 knowledge” OR “Social Interaction*” OR “Social Practice*” OR “social norms” OR “sharing NEAR/3 stor*” OR “Healthcare NEAR/3 communication” OR “healthcare NEAR/3 conversation*” OR “healthcare NEAR/3 information” OR “information NEAR/3 shar*” OR “information NEAR/3 flow*” OR “diffusion NEAR/3 information”

NOT (Doctor* or Dentist* or Nurse* or Clinician* or Professional* or Practitioner* or Healthcare professional*)

**AND**

“Health* service*” OR “Healthcare” OR “Health care” OR “Primary care” OR “Secondary care” OR “General practice” OR “Dental care” OR “Dental service*” OR “Oral health* Service*” OR “tertiary care” OR “advanced care” OR “mental health* service*” OR “Hospital*” OR “Doctor*” OR “Dentist*” OR “Physician*”

AND

[B]:

“Access*” OR “utilisation” OR “utilisation” OR “uptake” OR Visit* OR “Patient Acceptance of Health Care” OR "Facilities and Services Utilisation" OR "health* practice*" OR “decision-making” OR “Intra-familial decision making” OR “Delivery of Health Care” OR “Health* delivery” OR "Appointment* OR Schedule* OR “navigat* NEAR/3 health care” OR navigat* NEAR/3 "health care"

OR (influenc* OR shap* OR impact* )) AND ("healthcare behavi*r*" OR "health care behavi*r*" )

**[3]. CINAHL**

“local communit*” OR “neighborhood*” OR “neighbourhood*” OR “Neighborhood Characteristics” OR “Famil*” OR Familism OR Familismo OR “Friend*” OR “Family systems theory” OR “Community support” OR “Peer” OR “Peer group*” OR “Peer influence” OR “Generation*” OR “Interpersonal relation*” OR “Intergenerational relation*” OR “Social network*” OR “community network*” OR “Community group*” OR “famil* relation*” OR “Social ties” OR “Social group*” OR “Parent*” OR “Mother*” OR “Mother child relation*” OR “Father-child relation*” OR “Group dynamic*” OR “Intergeneration* N3 relation*” OR “Generation N3 relation*

AND

“knowledge construct*” OR “knowledge co-construct*” OR “knowledge co-produc*” OR “knowledge creation” OR “knowledge co-creation” OR “knowledge exchange” OR “Knowledge shar*” OR “Health knowledge” OR “Experiential knowledge” OR “narrative knowledge” OR “lay knowledge” OR “local knowledge” OR “Tacit knowledge” OR “indigenous knowledge” OR “Personal knowledge” OR “Traditional knowledge” OR “Familial knowledge” OR “Inherited knowledge” OR “Intergenerational knowledge” OR “knowledge dissemination” OR “perceived knowledge” OR “Lived experience*” OR "Shared experience*" OR "healthcare experience*" OR "health care experience*" OR “Storytelling” OR "story telling" OR "Story building" OR “sharing N3 stor*” OR “storytelling intervention” OR “Narration” OR “narrative intervention” OR “healthcare belief*” OR “healthcare N3 conversation*” OR “discussion network*” OR "community health literacy" OR “distributed health literacy” OR “health literacy as a social practice” OR “information N3 shar*” OR “Health* Information Exchange” OR “Health* information network*” OR “information N3 flow*” OR “diffusion N3 information” OR “healthcare N3 information” OR “healthcare advice” OR “Health* bricolage” OR “Personal narrative*” OR "knowledge practice*" OR “Community N4 Knowledge” OR “family N4 knowledge” OR “friends N4 knowledge” OR “intergenerational N4 knowledge” OR “Social Interaction*” OR “Social Practice*” OR “social norms” OR “Healthcare communication” NOT (Doctor* OR Dentist* OR Nurse* OR Clinician* OR Professional* OR Practitioner* OR "Healthcare professional*")

**AND**

[A]:

“Health* service*” OR “Health services needs and demand” OR “Community mental health service*” OR “Maternal health services” OR “Healthcare” OR “Health care” OR “Primary care” OR “Secondary health care” OR “General practice” OR “Dental care” OR “Dental service*” OR “Oral health* service*” OR “tertiary care” OR “advanced care” OR “Hospital*” OR “Doctor*” OR “Dentist*” OR “Physician*”

AND

[B]:

“Access*” OR “Access to information” OR “utilisation” OR “utilisation” OR “uptake” OR Visit* OR “Patient Acceptance of Health Care” OR "Facilities and Services Utilisation" OR "influence* health care" OR “health* decision-making” OR “Intra familial decision-making” OR “Delivery of Health Care” OR "Appointments and Schedules" OR “navigat* N3 health care” OR “navigat* N3 healthcare”

OR (((influenc* OR shap* OR impact*))) AND ("healthcare behavi*r*" OR "health care behavi*r*" )))

**[4].APA PsycInfo:**

“local communit*” OR “neighborhood*” OR “neighbourhood*” OR “Neighborhood Characteristics” OR “Famil*” OR Familism OR Familismo OR “Friend*” OR “Family systems theory” OR “Community support” OR “Peer” OR “Peer group*” OR “Peer influence” OR “Generation*” OR “Interpersonal relation*” OR “Intergenerational relation*” OR “Social network*” OR “community network*” OR “Community group*” OR “famil* relation*” OR “Social ties” OR “Social group*” OR “Social connectedness” OR “Parent*” OR “Mother*” OR “Mother child relation*” OR “Father-child relation*” OR “Group dynamic*” OR “Intergeneration* N3 relation*” OR “Generation N3 relation*

**AND**

“knowledge construct*” OR “knowledge co-construct*” OR “knowledge co-produc*” OR “knowledge creation” OR “knowledge co-creation” OR “knowledge exchange” OR “Knowledge shar*” OR “Knowledge transfer” OR “Health* knowledge” OR “Experiential knowledge” OR “narrative knowledge” OR “lay knowledge” OR “local knowledge” OR “Tacit knowledge” OR “indigenous knowledge” OR “Personal knowledge” OR “Traditional knowledge” OR “Familial knowledge” OR “Inherited knowledge” OR “Intergenerational knowledge” OR “knowledge dissemination” OR “perceived knowledge” OR “Lived experience*” OR "Shared experience*" OR "healthcare experience*" OR "health care experience*" OR “Storytelling” OR "story telling" OR "Story building" OR “sharing N3 stor*” OR “storytelling intervention” OR “Narration” OR “narrative intervention” OR “healthcare belief*” OR “Health belief model” OR “healthcare N3 conversation*” OR “discussion network*” OR “Group discussion” OR "community health literacy" OR “distributed health literacy” OR “health literacy as a social practice” OR “information N3 shar*” OR “Health* Information Exchange” OR “Health* information network*” OR “information N3 flow*” OR “diffusion N3 information” OR “healthcare N3 information” OR “healthcare advice” OR “Health* bricolage” OR “Personal narrative*” OR "knowledge practice*" OR “Community N4 Knowledge” OR “family N4 knowledge” OR “friends N4 knowledge” OR “intergenerational N4 knowledge” OR “Social Interaction*” OR “Social Practice*” OR “social norms” OR “Healthcare communication” NOT ( Doctor* OR Nurs* OR Professional* OR Dentist* OR "Healthcare professional*" OR Practitioner* )

**AND**

[A]:

“Health* service*” OR “Health services needs and demand” OR “Community mental health service*” OR “Maternal health services” OR “Healthcare” OR “Health care” OR “Primary care” OR “Secondary health care” OR “General practice” OR “Dental care” OR “Dental service*” OR “Oral health* service*” OR “tertiary care” OR “advanced care” OR “Hospital*” OR “Doctor*” OR “Dentist*” OR “Physician*”

**AND**

[B]:

“Access*” OR “Access to information” OR “utilisation” OR “utilisation” OR “uptake” OR Visit* OR “Patient Acceptance of Health Care” OR "Facilities and Services Utilisation" OR "influence* health care" OR “health* decision-making” OR “Intra familial decision-making” OR “Delivery of Health Care” OR "Appointments and Schedules" OR “navigat* N3 health care” OR “navigat* N3 healthcare”

OR (((influenc* OR shap* OR impact*))) AND ("Health care behaviour*" OR "Health care behavior*" OR "Healthcare behavior*" OR "Healthcare behaviour*") OR (DE "Health Care Utilization" OR DE "Health Care Access")
